# Supplementary material for: Emergent hypernetworks in weakly coupled oscillators
Source: Nat Commun. 2022 Aug 17;13:4849. doi: 10.1038/s41467-022-32282-4 (PMC9385626; doi:10.1038/s41467-022-32282-4)
Supplement: Supplementary file 1 — Supplementary Information [file 41467_2022_32282_MOESM1_ESM.pdf]

Supplementary Information to
Emergent hypernetworks in weakly coupled oscillators

Eddie Nijholt[1], Jorge Luis Ocampo-Espindola[2], Deniz Eroglu[3],
István Z. Kiss[2], Tiago Pereira[1,4*]

[1]Instituto de Ciências Matemáticas e Computação, Universidade de São Paulo, São Carlos, Brazil

[2]Department of Chemistry, Saint Louis University, St. Louis, USA

[3] Faculty of Engineering and Natural Sciences, Kadir Has University, Istanbul, Turkey

[4] Department of Mathematics, Imperial College London, SW7 2AZ, London, United Kingdom

Supplementary Notes

Supplementary Note 1: Experimental setup and methods **3**

SN 1.1 Experimental setup 3

SN 1.2 Behavior without coupling 3

SN 1.3 Phase dynamics 3

Supplementary Note 2: Proof of emergent higher-order networks **5**

SN 2.1 Preliminaries and results 6

SN 2.2 A coordinate transformation 15

SN 2.3 The first reduction 21

SN 2.4 The second reduction 23

Supplementary Note 3: Anomalous synchronization on a 4-node ring **27**

SN 3.1 Explanation of anomalous synchronization via normal form theory 28

Supplementary Note 4: Phase reduction for $h = (z^2+z)\bar{w}$ and resonance $\omega_1-\omega_{2,4}+\omega_3 = 0$ **30**

Supplementary Note 5: Slow phase dynamics for $h = (z^2 + z)\bar{w}$ and resonance $\omega_1 - \omega_{2,4} + \omega_3 = 0$ **30**

SN 5.1 Emergent hypernetwork predicts data behaviour 33

Supplementary Note 6: Model recovery of a 3-path with coupling $h = (z^2 + z)\bar{w}$ and resonance $\omega_1 - \omega_2 + \omega_3 = 0$ **33**

SN 6.1 Emergent network explanation 34

Supplementary Note 7: 6 nodes network examples with $h(z, w) = z\bar{w}$ **35**

Supplementary Note 8: Model recovery and normal form representation **37**

Supplementary Note 9: Emergent hypernetworks in an integrate-and-fire model **41**

Supplementary Note 10: Mean field interaction **42**
SN 10.1 Frequency shifts 44
SN 10.2 Model Recovery 44
SN 10.3 Normal Form Calculations 45

Supplementary Note

Supplementary Note 1: Experimental setup and methods

In this section, we describe the details about the experimental setup, the dynamical behavior of the oscillators without coupling, the of the phase model using LASSO.

SN 1.1 Experimental setup

The experiments were carried out in a standard three-electrode electrochemical cell. The cell consists of a nickel-array working electrode (W), a Pt-coated Ti rod as a counter electrode (C), and a Hg/Hg_2SO_4 sat. K_2SO_4 as a reference electrode (R). The electrolyte was a 3.0 M sulfuric acid solution at a constant temperature of 10 °C. The electrode array consisted of four 1-mm diameter nickel wires embedded in epoxy with a spacing of 3 mm. A multichannel potentiostat (Gill-IK64, ACM Instruments) interfaced with a real-time LabVIEW controller measured the potential drop $[E_k(t)$, with respect to the reference electrode] and set the circuit potential ($V_{0,k}$) of the working electrodes individually at a rate of 200 Hz. The electrode potentials are corrected for offset o_1=0.92 V, o_2=0.98 V, o_3= 0.91 V, and o_4=0.97 V.

SN 1.2 Behavior without coupling

The offset circuit potential to each oscillator was established 20 mV above the Hopf bifurcation ($V_{0,1}$=1850 mV, $V_{0,2}$=1100 mV, $V_{0,3}$=1660 mV, $V_{0,4}$=1103 mV). The natural frequencies [Supplementary Fig. 1 (a)] were adjusted to have values of ω_1=0.152 Hz, ω_2=0.385 Hz, ω_3=0.237 Hz and ω_4=0.384 Hz with a set of resistors and capacitors $R_{ind,1}$=12.0 kohm, C_{ind}=440 μF, $R_{ind,2}$=1.00 kohm, $R_{ind,3}$=12.0 kohm, $R_{ind,4}$=1.00 kohm. Without coupling, we observed that the slow oscillators (1 and 3) have about twice the amplitude than the fast oscillators (2 and 4). Supplementary Fig. 1 (c) shows the electrode potential time series of each oscillator.

SN 1.3 Phase dynamics

Phase definition. We used the peak-finding approach [1] to extract the phase of each oscillator and then linear interpolation between peaks from the experimental electrode potential time series. When there is no coupling, the pairwise phase difference shows a linear growth [Supplementary Fig.. 1 (b)] and the triplet phase differences, ϕ_j, j=1, 2, do not show phase slip behavior [Supplementary Fig. 1 (d)].

Fitting of phase dynamics As described in the main text, the impact of triplet interactions on the

Table 1: Recovered coefficients from Eq. 1.

Coefficients	Oscillator number			
	1	2	3	4
$\hat{\omega}_k^0$	0.953	2.368	1.467	2.383
$\hat{\omega}_k^1$	2.76×10^{-5}	0	-4.61×10^{-5}	0
$\hat{\omega}_k^2$	-6.40×10^{-8}	-6.63×10^{-8}	-1.32×10^{-7}	0
C_1^k	4.89×10^{-3}	5.04×10^{-3}	3.15×10^{-3}	0
D_1^k	9.27×10^{-5}	1.23×10^{-2}	-3.10×10^{-3}	0
C_2^k	-1.49×10^{-3}	0	3.16×10^{-3}	4.63×10^{-3}
D_2^k	-1.73×10^{-3}	0	-7.37×10^{-4}	1.64×10^{-2}

dynamics can be extracted with a LASSO fit to the $\dot{\theta}_k$ values according to

$$\dot{\theta}_k = \hat{\omega}_k(t) + \sum_{j=1}^{2} C_j^k \sin(\phi_j) + D_j^k \cos(\phi_j) \tag{1}$$

where $\hat{\omega}_k(t) = \hat{\omega}_k^0 + \hat{\omega}_k^1 t + \hat{\omega}_k^2 t^2$ is the fitted, slowly drifting (up to quadratic variation in time) natural frequency, and C_j^k and D_j^k are the amplitudes of the sin and cos phase coupling functions corresponding to the appropriate triplet phase differences.

For the fit, the instantaneous frequency, $\dot{\theta}_k$ was obtained with the numerical derivative of the phase of each oscillator from the experimental times series. The $\dot{\theta}_k$ was filtered by a first order Savitzky-Golay filter for 45 s. Using ϕ_j, we fitted the $\dot{\theta}_k$ with LASSO method. In LASSO, the regularization parameter determines how many parameters in the fitted model should be set to zero. We used a regularization parameter so that the mean square error is 20% higher than the best fit (no regularization). The fitted parameters are:

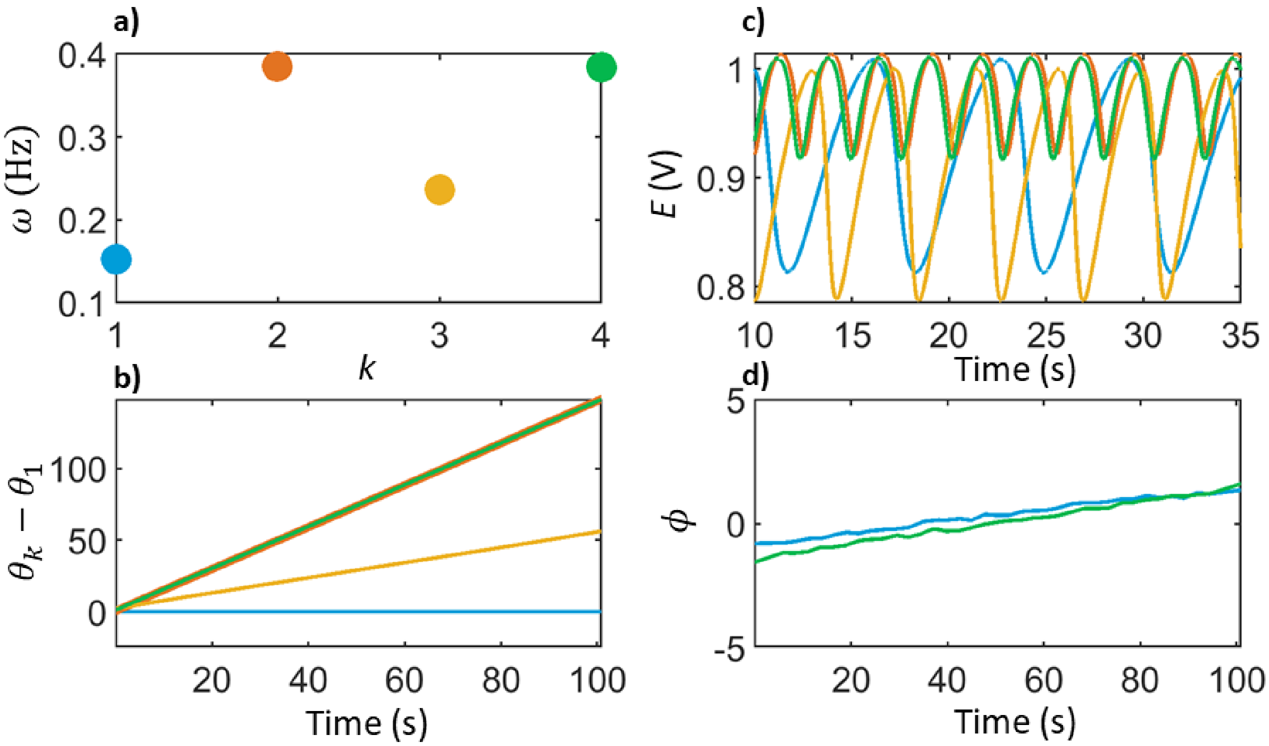

Supplementary Figure 1: **Dynamical behavior without coupling in the experiments.** a) Natural frequency of each oscillators without coupling ω_1=0.152 Hz, ω_2=0.385 Hz, ω_3=0.237 Hz and ω_4=0.384 Hz. The blue, orange, yellow and green dots represent the elements 1 to 4 respectively. b) Time series of the phase difference with respect oscillator one. The blue line: θ_1-$\theta_1 = 0$, orange line: θ_2-θ_1, yellow line: θ_3-θ_1 and green line: θ_4-θ_1. c) Electrode potential time series. Blue, orange, yellow, and green line corresponds to oscillator one to four respectively. d) Time series of the slow phases, ϕ_1 (blue) and ϕ_2 (green) without coupling.

Supplementary Note 2: Proof of emergent higher-order networks

We consider ODEs of the general form

$$\dot{z}_k = \gamma_k z_k - \beta_k z_k |z_k|^2 + \alpha H_k(z_1, \ldots, z_n)\,, \tag{2}$$

for $k \in \{1, \ldots, n\}$. Here, each z_k takes values in $\mathbb{C}$ and $\alpha \in \mathbb{R}$ denotes the coupling parameter of the interaction. We moreover have $\beta_k, \gamma_k \in \mathbb{C}$ non-zero, and write $\gamma_k = \lambda + i\omega_k$ for all $k \in \{1, \ldots, n\}$. Note that $\lambda \in \mathbb{R}$ may be seen as the bifurcation parameter for a Hopf bifurcation, which might in particular vanish. Each interaction function $H_k : \mathbb{C}^n \to \mathbb{C}$ is assumed smooth (i.e. C^∞) for convenience, and satisfies $H_k(0) = 0$ and $DH_k(0) = 0$.

We will show that the ODE (2) can be put in a particular normal form that allows us to predict

the dynamics of the phases of the oscillators. Our technique for doing so involves two successive coordinate transformations:

$$\begin{aligned} w_k &= z_k - \alpha P_k(z) \\ u_k &= w_k - \alpha Q_k(w) \end{aligned} \tag{3}$$

for some appropriately chosen polynomials P_k and Q_k. The first of these transformations is used to remove the term $\alpha H_k(z)$ from the ODE (2). This will generate additional terms in α^2 that may be expressed in the coefficients of H_k and P_k following certain combinatorial rules. In order to describe this combinatorial behavior, we first introduce a useful bracket $[\bullet||\bullet]$ on the space of polynomials, see Definition Supplementary Note 2:.6. The first coordinate transformation will also produce terms in α involving P_k and $\beta_k z_k |z_k|^2$. Again our bracket allows for a precise description of these new terms, which we then remove using the second coordinate transformation. The precise bookkeeping enabled by the bracket will be crucial in determining what non-resonance conditions are needed for the second transformation. In fact, it will turn out that the non-resonance conditions needed for the first transformation are sufficient to ensure the second.

We first present the main result, Theorem Supplementary Note 2:.4, in Subsection SN 2.1. There we also develop the necessary definitions, notation and machinery needed for the proof, which is then presented in the remaining subsections.

SN 2.1 Preliminaries and results

In order to analyse the ODE (2), it will be useful to write

$$H_k(z) = H_k^d(z) + \mathscr{O}(|z|^{d+1}),$$

where $H_k^d(z)$ is a polynomial denoting the terms up to degree d in the Taylor expansion of $H_k(z)$ around the origin. We will mostly work with the value $d = 5$. Note that $H_k^d(z)$ is therefore a polynomial in both the variables $z_1, \dots, z_n$ and their complex conjugates $\overline{z}_1, \dots, \overline{z}_n$, with complex coefficients. In general, whenever we talk about a polynomial we will always mean a complex polynomial in its given complex variables and their complex conjugates. It will also be useful to write $H(z) = (H_k(z))$ for the vector valued function that captures all interaction functions $H_k(z)$ as its components, and similarly set $H^d(z) = (H_k^d(z))$.

As is often the case with normal form calculations, we will need to assume some conditions on the ω_k (or more precisely the γ_k). These will depend on the coefficients of $H_k^5(z)$. To this end, we define:

Definition Supplementary Note 2:.1. Let

$$R(z) = c z_1^{s_1} \dots z_n^{s_n} \overline{z}_1^{t_1} \dots \overline{z}_n^{t_n} \tag{4}$$

be a monomial term in $H_k^d(z)$, where c is a complex number and $s_1, \dots, s_n, t_1, \dots, t_n$ are non-negative integers. The kth *non-resonance condition* of $R(z)$ is the condition

$$s_1\omega_1 + \cdots + s_n\omega_n - t_1\omega_1 - \cdots - t_n\omega_n - \omega_k \neq 0\,. \tag{5}$$

Note that, as ω_ℓ denotes the imaginary part of γ_ℓ for all $\ell \in \{1, \ldots, n\}$, the kth non-resonance condition guarantees in particular that:

$$s_1\gamma_1 + \cdots + s_n\gamma_n + t_1\overline{\gamma}_1 + \cdots + t_n\overline{\gamma}_n - \gamma_k \neq 0\,, \tag{6}$$

which will play a role in much of the proofs and constructions below. In fact, varying λ and allowing the particular case $\lambda = 0$, we see that equations (5) and (6) are equivalent in general. Next, the kth *non-resonance condition* of a polynomial is defined as the union of the kth non-resonance conditions of all of its monomial terms. Finally, the *non-resonance condition* of a polynomial map $H^d(z) = (H^d_k(z))$ is the union over all $k \in \{1, \ldots, n\}$ of the kth non-resonance conditions of $H^d_k(z)$. △

Example Supplementary Note 2:.2. Suppose the interaction functions are given by the polynomials

$$H_k(z) = \sum_{\ell=1}^{n} c_{k,\ell} z_k \overline{z}_\ell\,, \tag{7}$$

for some (possibly weighted) connection matrix $c = (c_{k,\ell}) \in \mathbb{C}^{n\times n}$. It follows that the kth non-resonance condition of $H_k(z)$ is given by

$$\omega_k - \omega_\ell - \omega_k = -\omega_\ell \neq 0 \quad \text{for all } \ell \text{ such that } c_{k,\ell} \neq 0\,. \tag{8}$$

Hence, we see that the non-resonance condition of $H(z) = (H_k(z))$ is satisfied if we simply have $\omega_\ell \neq 0$ for all nodes $\ell \in \{1, \ldots, n\}$. △

Example Supplementary Note 2:.3. Suppose the interaction functions are given by the polynomials

$$H_k(z) = \sum_{\ell=1}^{n} c_{k,\ell}(z_k\overline{z}_\ell + z_k^2\overline{z}_\ell)\,, \tag{9}$$

for some connection matrix $c = (c_{k,\ell})$. The kth non-resonance condition of $H_k(z)$ is now given by

$$\begin{aligned} \omega_k - \omega_\ell - \omega_k = -\omega_\ell \neq 0 \quad &\text{and} \\ 2\omega_k - \omega_\ell - \omega_k = \omega_k - \omega_\ell \neq 0 & \end{aligned} \tag{10}$$

for all ℓ such that $c_{k,l} \neq 0$. If we assume for convenience that c encodes a symmetric, connected graph, then the non-resonance condition of $H(z)$ is satisfied if

$$\begin{aligned} \omega_\ell \neq 0 \text{ for all nodes } \ell \in \{1, \ldots, n\} \text{ and} \\ \omega_p - \omega_q \neq 0 \text{ for all edges } e = [p, q] \text{ between nodes } p \text{ and } q\,. \end{aligned} \tag{11}$$

△

We are now ready to formulate the main theorem. It tells us that, under the relevant non-resonance conditions, we may transform the ODE (2) into a system with leading interaction terms involving only three-way "hyper-interactions" and with coupling constant α^2. See Proposition Supplementary Note 2:.9 for an exact description of the new leading interaction terms in Theorem Supplementary Note 2:.4.

Theorem Supplementary Note 2:.4. *Let H_k^5 denote the fifth order Taylor expansion of the kth interaction function $H_k : \mathbb{C}^n \to \mathbb{C}$. Assume the non-resonance conditions for $H^5 = (H_k^5)$ to hold. Then the ODE* (2) *is locally conjugate to*

$$\begin{aligned}\dot{u}_k = \gamma_k u_k - \beta_k u_k |u_k|^2 - \alpha^2 G_k(u) \\ + \mathscr{O}(|\alpha||u|^6 + |\alpha|^2|u|^5 + |\alpha|^3|u|^4)\,,\end{aligned} \tag{12}$$

with $u_k \in \mathbb{C}$ and for some complex polynomials G_k with only terms of degree 3 *and higher. See Proposition Supplementary Note 2:.9 for an exact description of the G_k.*

Note that Equation (12) gives a precise description of $\dot{u}_k$ up to sixth order in u and α.

Remark Supplementary Note 2:.5. Theorem Supplementary Note 2:.4 tells us that, under the relevant non-resonance conditions, the α-terms can be 'pushed back' to order $|\alpha||u|^6$. Technically speaking, this means we can ignore these remainder terms only when $\alpha u^6 << \alpha^2 u^4$, i.e. when $u^2 << \alpha$. However, it will be clear from the proof in subsections SN 2.2 and SN 2.4 that if the non-resonance conditions hold for terms in H_k beyond H_k^5, then we may arrange for a remainder in α with higher order terms in u. That is, we then get the remainder $\mathscr{O}(|\alpha||u|^N + |\alpha|^2|u|^5 + |\alpha|^3|u|^4)$ for a corresponding value of $N > 6$. As the interaction functions H_k are taken to be polynomials in our examples, with the non-resonance conditions holding for all terms, we in fact expect a remainder of the form $\mathscr{O}(|\alpha||u|^N + |\alpha|^2|u|^5 + |\alpha|^3|u|^4)$ for arbitrarily high value of N. Hence, we may neglect all terms that are first order in α, and obtain a new coupled system with coupling constant α^2. △

The new interaction functions G_k can be obtained from H by a combinatorial construction on the Taylor coefficients. This is best described using a bracket on polynomials that we define below. We will furthermore make extensive use of this bracket throughout the proof of Theorem Supplementary Note 2:.4.

Definition Supplementary Note 2:.6. Let $R(z)$ be a complex polynomial and let $S(z) = (S_1(z), \ldots, S_n(z))$ be an n-tuple (i.e., a vector) of complex polynomials $S_1(z), \ldots, S_n(z)$. We let $[R||S](z)$ be the complex polynomial obtained by (formally) assuming each variable z_j is time-dependent (i.e., $z_j = z_j(t)$) and satisfies $\dot{z}_j = S_j(z)$, after which we differentiate $R(z)$ with respect to t. That is, we set

$$[R||S](z) := \frac{d}{dt}R(z)\Bigg|\begin{array}{l}\dot{z}_j = S_j(w) \\ \forall j \in \\ \{1, \ldots, n\}\end{array}\,. \tag{13}$$

The reason we choose this notation, instead of one involving the Jacobian of R, is to avoid confusion with the complex conjugate variables $\overline{z}_j$. Because each term $\dot{\overline{z}}_j$ is substituted by $\overline{S_j(w)}$, the expression

$[R||S]$ is in general not complex linear in S.

In the special case where $S(z) = (\gamma_1 z_1, \ldots, \gamma_n z_n)$ (with γ_k as in Equation (2)), we set

$$\Gamma R(z) := [R||S](z) = \frac{d}{dt}R(z)\Bigg|_{\substack{\dot{z}_j = \gamma_j z_j \\ \forall j \in \\ \{1,\ldots,n\}}} . \tag{14}$$

△

Example Supplementary Note 2:.7. It is not hard to see that the polynomial $\Gamma R(z)$ is obtained by replacing every monomial

$$c z_1^{s_1} \ldots z_n^{s_n} \overline{z}_1^{t_1} \ldots \overline{z}_n^{t_n} , \quad c \in \mathbb{C}$$

in $R(z)$ by

$$c(s_1\gamma_1 + \cdots + s_n\gamma_n + t_1\overline{\gamma}_1 + \cdots + t_n\overline{\gamma}_n) z_1^{s_1} \ldots z_n^{s_n} \overline{z}_1^{t_1} \ldots \overline{z}_n^{t_n} .$$

Consider for instance the monomial $R(z) = R(z_1, z_2, \overline{z}_1, \overline{z}_2) = z_1^2 \overline{z}_2$. We have

$$\frac{d}{dt}R(z) = 2z_1\dot{z}_1\overline{z}_2 + z_1^2\dot{\overline{z}}_2 . \tag{15}$$

Hence, we indeed find

$$\begin{aligned}\Gamma R(z) = \frac{d}{dt}R(z)\Bigg|_{\substack{\dot{z}_1 = \gamma_1 z_1 \\ \dot{z}_2 = \gamma_2 z_2}} &= 2z_1(\gamma_1 z_1)\overline{z}_2 + z_1^2(\overline{\gamma_2 z_2}) \\ &= (2\gamma_1 + \overline{\gamma}_2) z_1^2 \overline{z}_2 = (2\gamma_1 + \overline{\gamma}_2) R(z) .\end{aligned} \tag{16}$$

△

The term G_k in Theorem Supplementary Note 2:.4 will be given as the bracket $[\bullet||\bullet]$ between H^5 and a polynomial obtained by slightly modifying H_k^5. Hence, intuitively, G_k should be thought of as $[H_k||H]$. More precisely, we define:

Definition Supplementary Note 2:.8. Let P be a polynomial for which its kth non-resonance conditions are met. The (kth) *modified polynomial* $\widehat{P}_k$ is obtained from P by replacing each monomial

$$c z_1^{s_1} \ldots z_n^{s_n} \overline{z}_1^{t_1} \ldots \overline{z}_n^{t_n} , \quad c \in \mathbb{C}$$

in P by

$$\frac{c z_1^{s_1} \ldots z_n^{s_n} \overline{z}_1^{t_1} \ldots \overline{z}_n^{t_n}}{s_1\gamma_1 + \cdots + s_n\gamma_n + t_1\overline{\gamma}_1 + \cdots + t_n\overline{\gamma}_n - \gamma_k} .$$

In the special case where $P = H_k^d$ for some $d \leq 5$, we will simply write $\widehat{H}_k^d := \widehat{(H_k^d)}_k$ to denote the corresponding modified polynomial. △

Proposition Supplementary Note 2:.9. *In Theorem Supplementary Note 2:.4 the terms G_k are given by*

$$G_k = [\widehat{H}_k^3 || H^3] \,. \tag{17}$$

It can be shown that $[\widehat{H}_k^3||H^3]$ indeed only has terms of degree 3 and higher, using the assumption that each H_k (and therefore each $\widehat{H}_k^d$) only has terms of degree 2 and higher. See Remark Supplementary Note 2:.15 below. Note that we only care about the third and fourth order terms of G_k, as the rest are absorbed in the remainder terms of Equation (12). It will be clear from Remark Supplementary Note 2:.15 that these lowest order terms do not change if we instead define

$$G_k = [\widehat{H}_k^d || H^d] \,, \tag{18}$$

for $d = 4$ or $d = 5$. For this reason we will often simply write

$$G_k = [\widehat{H}_k || H] \,. \tag{19}$$

Remark Supplementary Note 2:.10. Let $I_k \subset \{1, \ldots, n\}$ denote the *input set* of a node $k \in \{1, \ldots, n\}$. That is, I_k denotes those nodes that influence k, or more precisely those nodes ℓ for which

$$\frac{\partial H_k(z)}{\partial z_\ell} \neq 0 \text{ or } \frac{\partial H_k(z)}{\partial \overline{z}_\ell} \neq 0 \,.$$

Note that I_k might not contain k itself. It follows that in general $G_k(u)$ depends on variables for nodes in the set

$$I_k^2 := \left(\bigcup_{\ell \in I_k} I_\ell \right) \cup I_k \,. \tag{20}$$

This is because $G_k = [\widehat{H}_k^3||H^3]$ is formed by replacing a term z_ℓ (or $\overline{z}_\ell$) in $\widehat{H}_k^3$ by H_ℓ^3 (or $\overline{H_\ell^3}$), and this is done for each $\ell \in I_k$. We have also used here that $\widehat{H}_k^3$ likewise only depends on the variables corresponding to nodes in I_k, or possibly a strict subset thereof.

In a similar way one sees that the third order terms of G_k (that is, its leading order terms) are given by 'triplet terms' $\tilde{u}_r\tilde{u}_s\tilde{u}_t$, where we have $r \in I_k$ and $s, t \in I_\ell$ for some $\ell \in I_k$. (Here each $\tilde{u}_p$ may independently denote u_p or its complex conjugate $\overline{u}_p$). See Figure 2 for a schematic depiction of these emergent triplet terms. Of course the specifics of $H_k(z)$ might put additional constraints on the terms that can show up in $G_k(u)$. △

Example Supplementary Note 2:.11. As in Example Supplementary Note 2:.2, let us make the particular choice for the interaction functions

$$H_k(z) = H_k^3(z) = \sum_{\ell=1}^{n} c_{k,\ell} z_k \overline{z}_\ell \,. \tag{21}$$

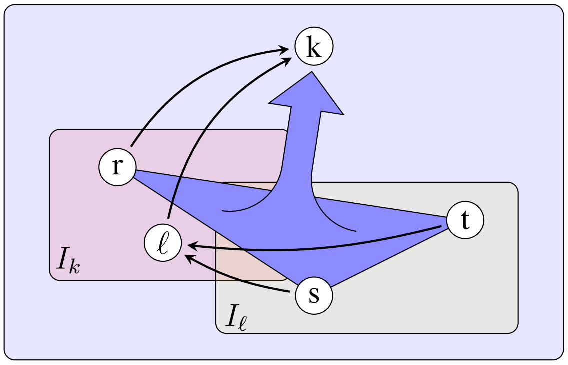

Supplementary Figure 2: Schematic depiction of the hidden 'triplet terms' that show up in G_k.

Here $c = (c_{k,\ell})$ is the connectivity matrix of the network. We will assume that $c_{k,\ell} \in \{0, 1\}$, or more generally $c_{k,\ell} \in \mathbb{R}$, if the network is weighted. We find

$$\widehat{H}_k(z) = \widehat{H}_k^3(z) = \sum_{\ell=1}^{n} \frac{c_{k,\ell}}{\overline{\gamma}_\ell} z_k \overline{z}_\ell \,. \tag{22}$$

A direct calculation now shows that

$$\begin{aligned} G_k(u) = [\widehat{H}_k||H](u) &= \sum_{\ell=1}^{n} \frac{c_{k,\ell}}{\overline{\gamma}_\ell} (H_k(u)\overline{u}_\ell + u_k \overline{H_\ell(u)}) \\ &= \sum_{\ell=1}^{n} \frac{c_{k,\ell}}{\overline{\gamma}_\ell} \left(\sum_{p=1}^{n} c_{k,p} u_k \overline{u}_p \overline{u}_\ell + u_k \sum_{p=1}^{n} c_{\ell,p} \overline{u}_\ell u_p \right) \\ &= \sum_{\ell=1}^{n} \sum_{p=1}^{n} \frac{c_{k,\ell} c_{k,p}}{\overline{\gamma}_\ell} u_k \overline{u}_\ell \overline{u}_p + \sum_{\ell=1}^{n} \sum_{p=1}^{n} \frac{c_{k,\ell} c_{\ell,p}}{\overline{\gamma}_\ell} u_k \overline{u}_\ell u_p \,. \end{aligned} \tag{23}$$

The first of these two terms counts all trees in the network that are formed by the node k and two nodes that influence node k. The second term counts all trees formed by the nodes k, ℓ and p, where k depends on ℓ and ℓ depends on p. △

We will gather some more facts about the bracket $[\bullet||\bullet]$. One important observation is given by:

Lemma Supplementary Note 2:.12. *The map $(R, S) \mapsto [R||S]$ is complex linear in R and real linear in S. In other words, given polynomials R, R' and complex numbers λ, μ, we have*

$$[\lambda R + \mu R'||S] = \lambda[R||S] + \mu[R'||S] \,. \tag{24}$$

Given tuples S, S' and real numbers a, b we have

$$[R||aS + bS'] = a[R||S] + b[R||S'] \,. \tag{25}$$

Proof. Complex linearity in R is clear from the definition:

$$[R||S](z) := \frac{d}{dt}R(z)\Bigg|\begin{array}{l}\dot{z}_j = S_j(w) \\ \forall j \in \\ \{1, \dots, n\}\end{array} . \tag{26}$$

Because of this, it suffices to show real linearity in S when R is given by a monomial of the form

$$R(z) = z_{i_1} z_{i_2} \dots z_{i_m} \overline{z}_{j_1} \overline{z}_{j_2} \dots \overline{z}_{j_l},$$

for some (not necessarily distinct) $i_1, \dots, i_m, j_1, \dots, j_l \in \{1, \dots, n\}$. We get

$$[R||S](z) = \sum_{s=1}^{m} \frac{R(z)}{z_{i_s}} S_{i_s}(z) + \sum_{r=1}^{l} \frac{R(z)}{\overline{z}_{i_r}} \overline{S_{i_r}(z)}, \tag{27}$$

from which real linearity in S follows readily. □

We will also make extensive use of the following definition:

Definition Supplementary Note 2:.13. Given a complex monomial

$$Q(z) = c z_1^{s_1} \dots z_n^{s_n} \overline{z}_1^{t_1} \dots \overline{z}_n^{t_n},$$

where $c \in \mathbb{C}$ and with $s_1, \dots, s_n, t_1, \dots, t_n$ non-negative integers, we define the degree of $Q(z)$ as the number $s_1 + \dots + s_n + t_1 + \dots + t_n$. The degree of a polynomial $P(z)$ is then defined as the maximum of the degrees of all the monomial terms of $P(z)$, as is common. Similarly, we define the *lower degree* of a polynomial $P(z)$ as the minimum of the degrees of all of its monomial terms. △

It turns out our bracket has a predictable effect on degrees:

Lemma Supplementary Note 2:.14. *Let R be a polynomial and $S = (S_1, \dots, S_n)$ a vector of polynomials. If R has degree $p > 0$ and each of the polynomial components of S has degree at most p', then $[R||S]$ has degree $p + p' - 1$ or lower. If R has lower degree $q > 0$ and each of the polynomial components of S has lower degree at least q', then $[R||S]$ has lower degree $q + q' - 1$ or higher.*

Proof. By linearity of the bracket (see Lemma Supplementary Note 2:.12), it suffices to show that the degree of $[R||S]$ is $d + d' - 1$ if R is a monomial of degree d and the components of S are all monomials of degree d'. (By convention, we treat the zero-polynomial as a polynomial of any degree.) As in the proof of Lemma Supplementary Note 2:.12, we write

$$R(z) = z_{i_1} z_{i_2} \dots z_{i_m} \overline{z}_{j_1} \overline{z}_{j_2} \dots \overline{z}_{j_l},$$

for some (not necessarily distinct) $i_1, \dots, i_m, j_1, \dots, j_l \in \{1, \dots, n\}$. It follows that $m + l = d$. As in the previous proof we find

$$[R||S](z) = \sum_{s=1}^{m} \frac{R(z)}{z_{i_s}} S_{i_s}(z) + \sum_{r=1}^{l} \frac{R(z)}{\overline{z}_{i_r}} \overline{S_{i_r}(z)}, \tag{28}$$

from which it follows readily that the degree of $[R||S]$ is indeed $d + d' - 1$. This completes the proof. □

Remark Supplementary Note 2:.15. Lemmas Supplementary Note 2:.12 and Supplementary Note 2:.14 give us an easy way of finding the third and fourth order terms of $G_k = [\widehat{H}_k||H]$ (and higher terms if necessary). It follows that the third order terms of G_k are given by $[\widehat{H}_k^2||H^2]$, where we use that H (and therefore each $\widehat{H}_k^2$) has no constant and linear terms. Likewise, the fourth order terms of G_k are given by the bracket between the second order terms of $\widehat{H}_k$ and the third order terms of H, plus the bracket between the third order terms of $\widehat{H}_k$ and the second order terms of H. Note also that G_k need only be defined up to third and fourth order in Theorem Supplementary Note 2:.4, as higher order terms of $\alpha^2 G_k(u)$ can be absorbed in the remainder $\mathscr{O}(|\alpha|^2|u|^5)$ of Equation (12). For this reason the terms of degree 4 and higher in $\widehat{H}_k$ and H play no role in the construction of (the relevant terms of) $G_k = [\widehat{H}_k||H]$. △

Next, we consider G_k in the situation where H_k describes a coupled cell system as in the examples of the main text.

Remark Supplementary Note 2:.16. Suppose each H_k is of the special form

$$H_k(z) = \sum_{\ell=1}^{n} c_{k,\ell} h(z_k, z_\ell) , \tag{29}$$

where $z = (z_1, \dots, z_n)$. Here $(c_{k,\ell})$ is a real adjacency matrix and $h : \mathbb{C}^2 \to \mathbb{C}$ has vanishing constant and linear terms. It follows that we may write

$$\widehat{H}_k(z) = \sum_{\ell=1}^{n} c_{k,\ell} \widehat{h}_{k,\ell}(z_k, z_\ell) , \tag{30}$$

where $\widehat{h}_{k,\ell}(z_k, z_\ell)$ is obtained from $h(z_k, z_\ell)$ by applying a monomial substitution to its terms in precisely the same way $\widehat{H}_k$ is obtained from H_k. By linearity of the bracket $[\bullet||\bullet]$ in the first slot, we get

$$G_k = [\widehat{H}_k||H] = [\sum_{\ell=1}^{n} c_{k,\ell} \widehat{h}_{k,\ell}(u_k, u_\ell)||H] = \sum_{\ell=1}^{n} c_{k,\ell} [\widehat{h}_{k,\ell}(u_k, u_\ell)||H] . \tag{31}$$

Moreover, we find

$$
\begin{aligned}
[\widehat{h}_{k,\ell}(u_k,u_\ell)||H](u) &= \frac{\partial \widehat{h}_{k,\ell}(u_k,u_\ell)}{\partial u_k} H_k + \frac{\partial \widehat{h}_{k,\ell}(u_k,u_\ell)}{\partial \overline{u}_k} \overline{H}_k + \frac{\partial \widehat{h}_{k,\ell}(u_k,u_\ell)}{\partial u_\ell} H_\ell + \frac{\partial \widehat{h}_{k,\ell}(u_k,u_\ell)}{\partial \overline{u}_\ell} \overline{H}_\ell \qquad (32) \\
&= \frac{\partial \widehat{h}_{k,\ell}(u_k,u_\ell)}{\partial u_k} \left(\sum_{p=1}^{n} c_{k,p} h(u_k,u_p) \right) + \frac{\partial \widehat{h}_{k,\ell}(u_k,u_\ell)}{\partial \overline{u}_k} \left(\sum_{p=1}^{n} c_{k,p} \overline{h(u_k,u_p)} \right) \\
&+ \frac{\partial \widehat{h}_{k,\ell}(u_k,u_\ell)}{\partial u_\ell} \left(\sum_{p=1}^{n} c_{\ell,p} h(u_\ell,u_p) \right) + \frac{\partial \widehat{h}_{k,\ell}(u_k,u_\ell)}{\partial \overline{u}_\ell} \left(\sum_{p=1}^{n} c_{\ell,p} \overline{h(u_\ell,u_p)} \right) \\
&= \sum_{p=1}^{n} c_{k,p} \left(\frac{\partial \widehat{h}_{k,\ell}(u_k,u_\ell)}{\partial u_k} h(u_k,u_p) + \frac{\partial \widehat{h}_{k,\ell}(u_k,u_\ell)}{\partial \overline{u}_k} \overline{h(u_k,u_p)} \right) \\
&+ \sum_{p=1}^{n} c_{\ell,p} \left(\frac{\partial \widehat{h}_{k,\ell}(u_k,u_\ell)}{\partial u_\ell} h(u_\ell,u_p) + \frac{\partial \widehat{h}_{k,\ell}(u_k,u_\ell)}{\partial \overline{u}_\ell} \overline{h(u_\ell,u_p)} \right) \\
&= \sum_{p=1}^{n} c_{k,p} \, {}^1G_k^{\ell p}(u_k,u_\ell,u_p) + \sum_{p=1}^{n} c_{\ell,p} \, {}^2G_k^{\ell p}(u_k,u_\ell,u_p) \, ,
\end{aligned}
$$

where we have set

$$
{}^1G_k^{\ell p}(u_k,u_\ell,u_p) := \frac{\partial \widehat{h}_{k,\ell}(u_k,u_\ell)}{\partial u_k} h(u_k,u_p) + \frac{\partial \widehat{h}_{k,\ell}(u_k,u_\ell)}{\partial \overline{u}_k} \overline{h(u_k,u_p)} \quad \text{and} \qquad (33)
$$

$$
{}^2G_k^{\ell p}(u_k,u_\ell,u_p) := \frac{\partial \widehat{h}_{k,\ell}(u_k,u_\ell)}{\partial u_\ell} h(u_\ell,u_p) + \frac{\partial \widehat{h}_{k,\ell}(u_k,u_\ell)}{\partial \overline{u}_\ell} \overline{h(u_\ell,u_p)} \, . \qquad (34)
$$

Combining equations (31) through (34), we obtain

$$
G_k(u) = \sum_{\ell=1}^{n} \sum_{p=1}^{n} c_{k,\ell} c_{k,p} \, {}^1G_k^{\ell p}(u_k,u_\ell,u_p) + \sum_{\ell=1}^{n} \sum_{p=1}^{n} c_{k,\ell} c_{\ell,p} \, {}^2G_k^{\ell p}(u_k,u_\ell,u_p) \, . \qquad (35)
$$

We may interpret Equation (35) as representing a new interaction structure, one where the interaction is now encoded through certain trees in the graph instead of links. In this regard, the emergent interaction function (35) looks a lot like our original response function (29), but counting such trees instead of links. The only way in which Equation (35) does not generalize Equation (29) perfectly is by the fact that ${}^1G_k^{\ell p}$ and ${}^1G_k^{\ell p}$ have indices k, ℓ and p (whereas h does not). However, we see from equations (33) and (34) that there is no dependence on p; this index is only there for notational purposes. Moreover, the dependence on k and ℓ is only through a rescaling of the monomials. Hence, we find an emergent interaction that is in very good agreement with a generalization of our original interaction to tree interaction. What is more, the trees that Equation (35) counts are easily identified in the original graph. See Example Supplementary Note 2:.11, which describes a special case of interaction through (29), and the corresponding Figure 3 of the main manuscript. △

Example Supplementary Note 2:.17. We return to Example Supplementary Note 2:.3, where the interaction functions are given by

$$H_k(z) = \sum_{\ell=1}^{n} c_{k,\ell}(z_k\overline{z}_\ell + z_k^2\overline{z}_\ell)\,. \tag{36}$$

This is of the form (29) as discussed in Remark Supplementary Note 2:.16, with h given by

$$h(z_k, z_\ell) = (z_k + z_k^2)\overline{z}_\ell\,. \tag{37}$$

Following the notation of Remark Supplementary Note 2:.16, we see that

$$\widehat{h}_{k,\ell}(z_k, z_\ell) = \frac{z_k\overline{z}_\ell}{\overline{\gamma}_\ell} + \frac{z_k^2\overline{z}_\ell}{\gamma_k + \overline{\gamma}_\ell}. \tag{38}$$

We therefore find

$$\begin{aligned} {}^1G_k^{\ell p}(u_k, u_\ell, u_p) &= \left(\frac{\overline{z}_\ell}{\overline{\gamma}_\ell} + \frac{2z_k\overline{z}_\ell}{\gamma_k + \overline{\gamma}_\ell}\right)(z_k + z_k^2)\overline{z}_p = \frac{(z_k + z_k^2)\overline{z}_\ell\overline{z}_p}{\overline{\gamma}_\ell} + \frac{2(z_k^2 + z_k^3)\overline{z}_\ell\overline{z}_p}{\gamma_k + \overline{\gamma}_\ell}\,, \\ {}^2G_k^{\ell p}(u_k, u_\ell, u_p) &= \left(\frac{z_k}{\overline{\gamma}_\ell} + \frac{z_k^2}{\gamma_k + \overline{\gamma}_\ell}\right)(\overline{z}_\ell + \overline{z}_\ell^2)z_p = \frac{z_k(\overline{z}_\ell + \overline{z}_\ell^2)z_p}{\overline{\gamma}_\ell} + \frac{z_k^2(\overline{z}_\ell + \overline{z}_\ell^2)z_p}{\gamma_k + \overline{\gamma}_\ell}\,. \end{aligned} \tag{39}$$

As we may ignore terms of degree 5 and higher, we may also set

$$\begin{aligned} {}^1G_k^{\ell p}(u_k, u_\ell, u_p) &= \frac{(z_k + z_k^2)\overline{z}_\ell\overline{z}_p}{\overline{\gamma}_\ell} + \frac{2z_k^2\overline{z}_\ell\overline{z}_p}{\gamma_k + \overline{\gamma}_\ell}\,, \\ {}^2G_k^{\ell p}(u_k, u_\ell, u_p) &= \frac{z_k(\overline{z}_\ell + \overline{z}_\ell^2)z_p}{\overline{\gamma}_\ell} + \frac{z_k^2\overline{z}_\ell z_p}{\gamma_k + \overline{\gamma}_\ell}\,, \end{aligned} \tag{40}$$

which describe the new interaction through Equation (35). △

SN 2.2 A coordinate transformation

In this subsection and the next ones we prove Theorem Supplementary Note 2:.4 and the accompanying Proposition Supplementary Note 2:.9. Recall that we want to transform

$$\dot{z}_k = \gamma_k z_k - \beta_k z_k|z_k|^2 + \alpha H_k(z)\,, \tag{41}$$

into an ODE where the leading interaction terms are of order α^2. Recall as well that $H_k^5(z)$ denotes the Taylor expansion of $H_k(z)$ up to fifth order. In particular, we may write

$$H_k(z) = H_k^5(z) + \mathscr{O}(|z|^6)\,. \tag{42}$$

It follows that $H_k^5(z)$ is a complex polynomial of order 5 in the variables $z_1, \dots, z_n$ and $\overline{z}_1, \dots, \overline{z}_n$. We write $z = (z_1, \dots, z_n)$, and similarly for other variables, and assume implicitly that any function of z may also depend on its complex conjugate $\overline{z} = (\overline{z}_1, \dots, \overline{z}_n)$.

We start by rewriting the ODE (41) using the transformation

$$w_k = z_k - \alpha \widehat{H}_k^5(z) = z_k - \alpha P_k(z)\,, \tag{43}$$

where we have set $P_k := \widehat{H}_k^5$ for convenience. Note that each $P_k : \mathbb{C}^n \to \mathbb{C}$ is a complex polynomial of lower degree 2 (see Definition Supplementary Note 2:.13). It follows that Expression (43) describes an invertible transformation around $z = 0$. The following lemma deals with its inverse.

Lemma Supplementary Note 2:.18. *Suppose the variables $w = (w_1, \ldots, w_n)$ may be expressed in $z = (z_1, \ldots, z_n)$ and α by*

$$w_k = z_k - \alpha P_k(z)\,, \tag{44}$$

for some polynomials P_k of lower degree $d \geq 2$. Then z can be expressed in w and α by the formal expression

$$z_k = w_k + \alpha P_k(w) + \alpha^2 R_{k,2}(w) + \alpha^3 R_{k,3}(w) + \ldots\,. \tag{45}$$

Here the $R_{k,t}(w)$ are polynomials with lower degree $(d-1)t + 1$ or higher.

Proof. We write

$$z_k = R_{k,0}(w) + \alpha R_{k,1}(w) + \alpha^2 R_{k,2}(w) + \ldots\,, \tag{46}$$

for some functions $R_{k,t}(w) : \mathbb{C}^n \to \mathbb{C}$. To determine these functions, we substitute the z variables in Equation (44) by Expression (46). We obtain

$$\begin{aligned} w_k &= z_k - \alpha P_k(z_1, \ldots, z_n) \qquad (47)\\ &= [R_{k,0}(w) + \alpha R_{k,1}(w) + \ldots] - \alpha P_k([R_{1,0}(w) + \alpha R_{1,1}(w) + \ldots], \ldots, [R_{n,0}(w) + \alpha R_{n,1}(w) + \ldots])\,. \end{aligned}$$

Comparing constant terms in α (i.e. α^0), Expression (47) gives us

$$w_k = R_{k,0}(w)\,. \tag{48}$$

This simplifies Equation (47) to

$$w_k = [w_k + \alpha R_{k,1}(w) + \ldots] - \alpha P_k([w_1 + \alpha R_{1,1}(w) + \ldots], \ldots, [w_n + \alpha R_{n,1}(w) + \ldots])\,. \tag{49}$$

Comparing α-terms now yields

$$0 = R_{k,1}(w) - P_k(w)\,, \tag{50}$$

so that

$$R_{k,1}(w) = P_k(w)\,. \tag{51}$$

It remains to show that the higher order terms are indeed polynomials of the required lower degree. We will show this by induction on t. Note that $R_{k,0}(w) = w_k$ has (lower) degree $(d-1)0 + 1 = 1$. Likewise, $R_{k,1}(w) = P_k(w)$ is of lower degree $(d-1)1 + 1 = d$. We therefore fix an integer $T > 1$ and assume that the function $R_{k,t}(w)$ is a complex polynomial of lower degree $(d-1)t + 1$ or higher for all $t < T$ and $k \in \{1, \ldots, n\}$. The α^T terms in Equation (49) are given by

$$0 = R_{k,T}(w) - [\alpha^{T-1}]P_k([w_1 + \alpha R_{1,1}(w) + \ldots], \ldots, [w_n + \alpha R_{n,1}(w) + \ldots])\,. \tag{52}$$

Here $[\alpha^{T-1}]F(\alpha)$ denotes the α^{T-1} term in the expansion of a function F in α. As P_k is a polynomial of lower degree d, the α^{T-1} term in

$$P_k([w_1 + \alpha R_{1,1}(w) + \dots], \dots, [w_n + \alpha R_{n,1}(w) + \dots])$$

must be a finite sum of scalar multiples of expressions of the form

$$\tilde{R}_{i_1,t_1}(w)\tilde{R}_{i_2,t_2}(w)\dots\tilde{R}_{i_s,t_s}(w)\,,$$

for $s \geq d$ and for some $i_1, \dots, i_s \in \{1, \dots, n\}$ and $t_1, \dots, t_s \in \mathbb{Z}_{\geq 0}$ satisfying $t_1 + \dots + t_s = T - 1$. Each term $\tilde{R}_{i_j,t_j}(w)$ may furthermore independently denote $R_{i_j,t_j}(w)$ or its complex conjugate $\overline{R_{i_j,t_j}(w)}$. As we have $t_1 + \dots + t_s = T - 1$, it in particular holds that $t_1, \dots, t_s \leq T - 1$. By the induction hypothesis, we therefore know that each of the terms $\tilde{R}_{i_j,t_j}$ is a polynomial of lower degree $(d-1)t_j + 1$ or higher. This means the expression

$$\tilde{R}_{i_1,t_1}(w)\tilde{R}_{i_2,t_2}(w)\dots\tilde{R}_{i_s,t_s}(w)$$

is a polynomial of lower degree D satisfying

$$\begin{aligned} D &\geq [(d-1)t_1 + 1] + [(d-1)t_2 + 1] + \dots + [(d-1)t_s + 1] \\ &= (d-1)(t_1 + t_2 + \dots + t_s) + s \\ &= (d-1)(T-1) + s \geq (d-1)(T-1) + d \\ &= (d-1)T + 1\,. \end{aligned}$$

It follows from Equation (52) that $R_{k,T}(w)$ is indeed a polynomial of lower degree $(d-1)T + 1$ or higher for all $k \in \{1, \dots, n\}$. This proves the lemma by induction. □

Setting $d = 2$, it follows that the inverse of Equation (43) is given by

$$z_k = w_k + \alpha P_k(w) + \mathscr{O}(|\alpha|^2|w|^3)\,. \tag{53}$$

At some point later on, we will need to know Expression (53) up to higher order terms. To this end, we will show how our bracket $[\bullet||\bullet]$ from Definition Supplementary Note 2:.6 shows up when performing coordinate transformations.

Lemma Supplementary Note 2:.19. *Let $R(z)$ be a complex polynomial and suppose we may express the z-variables in some new w-variables by*

$$z_k = w_k + \alpha S_k(w) + \mathscr{O}(|\alpha|^2)\,, \quad k \in \{1, \dots, n\}\,. \tag{54}$$

Here each S_k is a complex polynomial and we have $\alpha \in \mathbb{R}$. Then $R(z)$ is given in the w-variables by

$$R(z) = R(w) + \alpha[R||S](w) + \mathscr{O}(|\alpha|^2)\,, \tag{55}$$

where we have set $S = (S_1, \dots, S_n)$.

Proof. We write $R(z)$ as

$$R(z) = T_0(w) + \alpha T_1(w) + \mathscr{O}(|\alpha|^2)\,, \tag{56}$$

where $T_0(w)$ and $T_1(w)$ are to be determined. Assume first that $R(z)$ is given by

$$R(z) = z_{i_1} z_{i_2} \dots z_{i_m} \overline{z}_{j_1} \overline{z}_{j_2} \dots \overline{z}_{j_l},$$

for some (not necessarily distinct) $i_1, \dots, i_m, j_1, \dots, j_l \in \{1, \dots, n\}$. We get

$$\begin{aligned} R(z) &= z_{i_1} \dots z_{i_m} \overline{z}_{j_1} \dots \overline{z}_{j_l} \\ &= (w_{i_1} + \alpha S_{i_1}(w)) \dots (w_{i_m} + \alpha S_{i_m}(w)) \overline{(w_{j_1} + \alpha S_{j_1}(w))} \dots \overline{(w_{j_l} + \alpha S_{j_l}(w))} + \mathscr{O}(|\alpha|^2) \\ &= w_{i_1} \dots w_{i_m} \overline{w}_{j_1} \dots \overline{w}_{j_l} + \alpha \left(\sum_{s=1}^{m} \frac{R(w)}{w_{i_s}} S_{i_s}(w) + \sum_{r=1}^{l} \frac{R(w)}{\overline{w}_{i_r}} \overline{S_{i_r}(w)} \right) + \mathscr{O}(|\alpha|^2) \\ &= R(w) + \alpha [R||S](w) + \mathscr{O}(|\alpha|^2)\,, \end{aligned} \tag{57}$$

where in the last line we have used Expression (27) from the proof of Lemma Supplementary Note 2:.12. As T_0 and T_1 are determined linearly by R, we may conclude from Lemma Supplementary Note 2:.12 that $T_0(w) = R(w)$ and $T_1(w) = [R||S](w)$ for general polynomials R. This completes the proof. □

Example Supplementary Note 2:.20. Suppose we are given the polynomial $Q(z) = Q(z_1, z_2, \overline{z}_1, \overline{z}_2) = z_1^2 + z_1 \overline{z}_2$. Equation (54) gives

$$\begin{aligned} Q(z) &= (w_1 + \alpha S_1(w) + \mathscr{O}(|\alpha|^2))^2 + (w_1 + \alpha S_1(w) + \mathscr{O}(|\alpha|^2) \overline{(w_2 + \alpha S_2(w) + \mathscr{O}(|\alpha|^2))} \\ &= (w_1 + \alpha S_1(w))^2 + (w_1 + \alpha S_1(w))(\overline{w}_2 + \alpha \overline{S_2(w)}) + \mathscr{O}(|\alpha|^2) \\ &= w_1^2 + w_1 \overline{w}_2 + \alpha (2 w_1 S_1(w) + w_1 \overline{S_2(w)} + S_1(w) \overline{w}_2) + \mathscr{O}(|\alpha|^2) \\ &= Q(w) + \alpha \frac{d}{dt} Q(w) \Bigg|_{\substack{\dot{w}_1 = S_1(w) \\ \dot{w}_2 = S_2(w)}} + \mathscr{O}(|\alpha|^2) \\ &= Q(w) + \alpha [Q||S](w) + \mathscr{O}(|\alpha|^2)\,, \end{aligned} \tag{58}$$

which is in accordance with Lemma Supplementary Note 2:.19. △

Returning to the transformation (43) with inverse Equation (53), we may in fact conclude the following:

Lemma Supplementary Note 2:.21. *Suppose we have a coordinate transformation of the form*

$$w_k = z_k - \alpha P_k(z)\,, \tag{59}$$

where each $P_k : \mathbb{C}^n \to \mathbb{C}$ is a complex polynomial of lower degree 2 or higher. The inverse transformation is given by

$$z_k = w_k + \alpha P_k(w) + \alpha^2 [P_k||P](w) + \mathscr{O}(|\alpha|^3 |w|^4)\,, \tag{60}$$

where $P = (P_1, \dots, P_n)$.

Proof. It follows from Lemma Supplementary Note 2:.18 that we may write

$$z_k = w_k + \alpha P_k(w) + \alpha^2 R_{k,2}(w) + \mathscr{O}(|\alpha|^3 |w|^4)\,, \tag{61}$$

for some function $R_{k,2}(w)$. Hence, we only have to show that $R_{k,2}(w) = [P_k||P](w)$. To this end, we rewrite Expression (59) as

$$z_k = w_k + \alpha P_k(z)\,. \tag{62}$$

Next, we use Equation (61) to write

$$z_k = w_k + \alpha P_k(w) + \mathscr{O}(|\alpha|^2)\,. \tag{63}$$

Applying Lemma Supplementary Note 2:.19 to the term $P_k(z)$ and the transformation (63) yields

$$P_k(z) = P_k(w) + \alpha[P_k||P](w) + \mathscr{O}(|\alpha|^2)\,. \tag{64}$$

Combined with Equation (62), we obtain

$$\begin{aligned} z_k &= w_k + \alpha(P_k(w) + \alpha[P_k||P](w) + \mathscr{O}(|\alpha|^2)) \\ &= w_k + \alpha P_k(w) + \alpha^2[P_k||P](w) + \mathscr{O}(|\alpha|^3)\,. \end{aligned} \tag{65}$$

Comparing the two expressions (61) and (65) for z_k, we see that indeed

$$z_k = w_k + \alpha P_k(w) + \alpha^2[P_k||P](w) + \mathscr{O}(|\alpha|^3 |w|^4)\,. \tag{66}$$

This proves the lemma. □

Our next step is to differentiate Equation (43) with respect to time. This gives us

$$\dot{w}_k = \dot{z}_k - \alpha \frac{d}{dt} P_k(z)\,. \tag{67}$$

We will first focus on the term

$$\frac{d}{dt} P_k(z)\,, \tag{68}$$

and then deal with the term $\dot{z}_k$.

The term $\partial_t P_k$ We first focus on the term (68). We start by rewriting Equation (41) as

$$\begin{aligned} \dot{z}_k &= \gamma_k z_k - \beta_k z_k |z_k|^2 + \alpha H_k(z) \\ &= \gamma_k z_k - \beta_k z_k |z_k|^2 + \alpha H_k^5(z) + \mathscr{O}(|\alpha||z|^6)\,, \end{aligned} \tag{69}$$

where we recall that $H_k^5(z)$ denotes the Taylor expansion of $H_k(z)$ up to fifth order. From Equation (69) and Lemma Supplementary Note 2:.12 we get

$$\begin{aligned}\frac{d}{dt}P_k(z) &= \frac{d}{dt}P_k(z)\Bigg|\begin{array}{l}\dot z_j = \gamma_j z_j - \beta_j z_j|z_j|^2 + \alpha H_j(z)\\ \forall\, j\in\{1,\dots,n\}\end{array} \\ &= \frac{d}{dt}P_k(z)\Bigg|\begin{array}{l}\dot z_j = \gamma_j z_j - \beta_j z_j|z_j|^2 + \alpha H_j^5(z)\\ \forall\, j\in\{1,\dots,n\}\end{array} \quad + \quad \mathscr{O}(|\alpha||z|^7) \\ &= [P_k||(\dots,\gamma_j z_j - \beta_j z_j|z_j|^2 + \alpha H_j^5(z),\dots)](z) + \mathscr{O}(|\alpha||z|^7) \\ &= [P_k||(\dots,\gamma_j z_j,\dots)](z) - [P_k||(\dots,\beta_j z_j|z_j|^2,\dots)](z) \\ &+ \alpha[P_k||(\dots,H_j^5(z),\dots)](z) + \mathscr{O}(|\alpha||z|^7) \\ &= \Gamma P_k(z) - L_k^1(z) + \alpha[P_k||H^5](z) + \mathscr{O}(|\alpha||z|^7)\,,\end{aligned} \tag{70}$$

where we have set

$$L_k^1(z) := [P_k||(\dots,\beta_j z_j|z_j|^2,\dots)](z)$$

and

$$H^5 := (\dots,H_j^5(z),\dots)\,.$$

We have moreover used that $P_k(z)$ has lower degree at least 2 to arrive at the remainder term $\mathscr{O}(|\alpha||z|^7)$, and we refer to Definition Supplementary Note 2:.6 for the meaning of the term $\Gamma P_k(z)$. Note that $L_k^1(z)$ is a polynomial of lower degree at least 4, whereas $[P_k||H^5](z)$ has lower degree 3 or higher.

Next, we return to Equation (53), which we recall states

$$z_k = w_k + \alpha P_k(w) + \mathscr{O}(|\alpha|^2|w|^3) = w_k + \mathscr{O}(|\alpha||w|^2)\,. \tag{71}$$

We obtain

$$L_k^1(z) = L_k^1(w) + \mathscr{O}(|\alpha||w|^5) \tag{72}$$

$$[P_k||H^5](z) = [P_k||H^5](w) + \mathscr{O}(|\alpha||w|^4)\,. \tag{73}$$

From Lemma Supplementary Note 2:.19 we furthermore get

$$\Gamma P_k(z) = \Gamma P_k(w) + \alpha[\Gamma P_k||P](w) + \mathscr{O}(|\alpha|^2|w|^4)\,, \tag{74}$$

where we have set $P = (P_1,\dots,P_n)$. The remainder term in Equation (74) follows from the lower degrees of $\Gamma P_k(z)$ and $P_k(z)$, and the remainder in Equation (71).

Note that $[\Gamma P_k||P](w)$ is a polynomial of lower degree 3 or higher. Combining equations (70), (72), (73) and (74), we arrive at:

Lemma Supplementary Note 2:.22. *The term*

$$\frac{d}{dt}P_k(z)$$

may be expressed in the new w coordinates by

$$\begin{aligned}\frac{d}{dt}P_k(z) &= \Gamma P_k(z) - L_k^1(z) + \alpha[P_k||H^5](z) + \mathscr{O}(|\alpha||z|^7) \\ &= \Gamma P_k(w) - L_k^1(w) + \alpha[P_k||H^5](w) + \alpha[\Gamma P_k||P](w) + \mathscr{O}(|\alpha||w|^5 + |\alpha|^2|w|^4)\,.\end{aligned} \tag{75}$$

The term $\dot{z}_k$ Next, we focus on the term $\dot{z}_k$. Again we write.

$$\begin{aligned}\dot{z}_k &= \gamma_k z_k - \beta_k z_k |z_k|^2 + \alpha H_k(z) \\ &= \gamma_k z_k - \beta_k z_k |z_k|^2 + \alpha H_k^5(z) + \mathscr{O}(|\alpha||z|^6)\,,\end{aligned} \tag{76}$$

where $H_k^5(z)$ denotes the Taylor expansion of $H_k(z)$ up to fifth order.

Recall the result of Lemma Supplementary Note 2:.21, which tells us that

$$z_k = w_k + \alpha P_k(w) + \alpha^2 [P_k||P](w) + \mathscr{O}(|\alpha|^3 |w|^4)\,. \tag{77}$$

Combined, and using Lemma Supplementary Note 2:.19, we get

$$\begin{aligned}\dot{z}_k &= \gamma_k z_k - \beta_k z_k |z_k|^2 + \alpha H_k^5(z) + \mathscr{O}(|\alpha||z|^6) \\ &= \gamma_k (w_k + \alpha P_k(w) + \alpha^2 [P_k||P](w)) + \mathscr{O}(|\alpha|^3 |w|^4) \\ &- \beta_k w_k |w_k|^2 - \alpha [\beta_k w_k |w_k|^2 ||P](w) + \mathscr{O}(|\alpha|^2 |w|^5) \\ &+ \alpha H_k^5(w) + \alpha^2 [H_k^5||P](w) + \mathscr{O}(|\alpha|^3 |w|^4) \\ &+ \mathscr{O}(|\alpha||w|^6) \\ &= \gamma_k w_k - \beta_k w_k |w_k|^2 + \alpha(\gamma_k P_k(w) + H_k^5(w)) - \alpha [\beta_k w_k |w_k|^2 ||P](w) \\ &+ \alpha^2 (\gamma_k [P_k||P](w) + [H_k^5||P](w)) + \mathscr{O}(|\alpha||w|^6 + |\alpha|^2 |w|^5 + |\alpha|^3 |w|^4)\,.\end{aligned} \tag{78}$$

We will write

$$L_k^2(w) := [\beta_k w_k |w_k|^2 ||P](w)\,, \tag{79}$$

which has lower degree 4 or higher, to arrive at:

Lemma Supplementary Note 2:.23. *The term $\dot{z}_k$ may be expressed in the new w coordinates by*

$$\begin{aligned}\dot{z}_k &= \gamma_k w_k - \beta_k w_k |w_k|^2 \\ &+ \alpha(\gamma_k P_k(w) + H_k^5(w)) - \alpha L_k^2(w) \\ &+ \alpha^2 (\gamma_k [P_k||P](w) + [H_k^5||P](w)) \\ &+ \mathscr{O}(|\alpha||w|^6 + |\alpha|^2 |w|^5 + |\alpha|^3 |w|^4)\,.\end{aligned} \tag{80}$$

SN 2.3 The first reduction

We may now substitute the results of Lemma Supplementary Note 2:.22 and Lemma Supplementary Note 2:.23 into

$$\dot{w}_i = \dot{z}_i - \alpha \frac{d}{dt} P_i(z)\,. \tag{81}$$

We obtain

$$
\begin{aligned}
\dot{w}_k &= \gamma_k w_k - \beta_k w_k |w_k|^2 \\
&\quad + \alpha(\gamma_k P_k(w) + H_k^5(w)) - \alpha L_k^2(w) \\
&\quad + \alpha^2(\gamma_k [P_k||P](w) + [H_k^5||P](w)) \\
&\quad + \mathscr{O}(|\alpha||w|^6 + |\alpha|^2|w|^5 + |\alpha|^3|w|^4) \\
&\quad - \alpha(\Gamma P_k(w) - L_k^1(w) + \alpha[P_k||H^5](w) + \alpha[\Gamma P_k||P](w)) \\
&= \gamma_k w_k - \beta_k w_k |w_k|^2 \\
&\quad + \alpha(\gamma_k P_k(w) + H_k^5(w) - \Gamma P_k(w)) + \alpha(L_k^1(w) - L_k^2(w)) \\
&\quad + \alpha^2(\gamma_k [P_k||P](w) + [H_k^5||P](w) - [P_k||H^5](w) - [\Gamma P_k||P](w)) \\
&\quad + \mathscr{O}(|\alpha||w|^6 + |\alpha|^2|w|^5 + |\alpha|^3|w|^4)\,.
\end{aligned} \tag{82}
$$

By Lemma Supplementary Note 2:.12 we may further write this as

$$
\begin{aligned}
\dot{w}_k &= \gamma_k w_k - \beta_k w_k |w_k|^2 \\
&\quad + \alpha(\gamma_k P_k(w) + H_k^5(w) - \Gamma P_k(w)) + \alpha(L_k^1(w) - L_k^2(w)) \\
&\quad + \alpha^2[\gamma_k P_k + H_k^5 - \Gamma P_k||P](w) - \alpha^2[P_k||H^5](w) \\
&\quad + \mathscr{O}(|\alpha||w|^6 + |\alpha|^2|w|^5 + |\alpha|^3|w|^4)\,.
\end{aligned} \tag{83}
$$

Next, we claim that our choice of polynomial $P_k = \widehat{H}_k^5$ guarantees that the term

$$\gamma_k P_k + H_k^5 - \Gamma P_k$$

vanishes. More precisely, we prove:

Lemma Supplementary Note 2:.24. *Let Q be a polynomial for which the kth non-resonance condition is satisfied. In particular, it follows that the corresponding modified polynomial $\widehat{Q}_k$ is well-defined. We then have*

$$\gamma_k \widehat{Q}_k + Q - \Gamma \widehat{Q}_k = 0\,. \tag{84}$$

Proof. By definitions Supplementary Note 2:.6 and Supplementary Note 2:.8, we see that it suffices to show this when Q is given by a single monomial

$$Q(z) = z_1^{s_1} \dots z_n^{s_n} \overline{z}_1^{t_1} \dots \overline{z}_n^{t_n}\,,$$

where $t_1, \dots, t_n, s_1, \dots, s_n$ are non-negative integers. More precisely, we use here that the maps $Q \mapsto \widehat{Q}_k$ and $Q \mapsto \Gamma Q = [Q|| \dots, \gamma_j z_j, \dots]$ are complex linear, when defined. By Definition Supplementary Note 2:.8 we find

$$\widehat{Q}_k(z) = \frac{z_1^{s_1} \dots z_n^{s_n} \overline{z}_1^{t_1} \dots \overline{z}_n^{t_n}}{s_1\gamma_1 + \dots + s_n\gamma_n + t_1\overline{\gamma}_1 + \dots + t_n\overline{\gamma}_n - \gamma_k} = \frac{Q(z)}{s_1\gamma_1 + \dots + s_n\gamma_n + t_1\overline{\gamma}_1 + \dots + t_n\overline{\gamma}_n - \gamma_k}\,.$$

Example Supplementary Note 2:.7 now tells us that

$$\Gamma\widehat{Q}_k(z) = \frac{(s_1\gamma_1 + \dots + s_n\gamma_n + t_1\overline{\gamma}_1 + \dots + t_n\overline{\gamma}_n) z_1^{s_1} \dots z_n^{s_n} \overline{z}_1^{t_1} \dots \overline{z}_n^{t_n}}{s_1\gamma_1 + \dots + s_n\gamma_n + t_1\overline{\gamma}_1 + \dots + t_n\overline{\gamma}_n - \gamma_k} = \frac{(s_1\gamma_1 + \dots + s_n\gamma_n + t_1\overline{\gamma}_1 + \dots + t_n\overline{\gamma}_n) Q(z)}{s_1\gamma_1 + \dots + s_n\gamma_n + t_1\overline{\gamma}_1 + \dots + t_n\overline{\gamma}_n - \gamma_k}. \tag{85}$$

We therefore conclude that

$$\begin{aligned}\Gamma\widehat{Q}_k(z) - \gamma_k\widehat{Q}_k(z) &= \frac{(s_1\gamma_1 + \dots + s_n\gamma_n + t_1\overline{\gamma}_1 + \dots + t_n\overline{\gamma}_n) Q(z)}{s_1\gamma_1 + \dots + s_n\gamma_n + t_1\overline{\gamma}_1 + \dots + t_n\overline{\gamma}_n - \gamma_k} \\ &\quad - \frac{\gamma_k Q(z)}{s_1\gamma_1 + \dots + s_n\gamma_n + t_1\overline{\gamma}_1 + \dots + t_n\overline{\gamma}_n - \gamma_k} \\ &= \frac{(s_1\gamma_1 + \dots + s_n\gamma_n + t_1\overline{\gamma}_1 + \dots + t_n\overline{\gamma}_n - \gamma_k) Q(z)}{s_1\gamma_1 + \dots + s_n\gamma_n + t_1\overline{\gamma}_1 + \dots + t_n\overline{\gamma}_n - \gamma_k} = Q(z).\end{aligned} \tag{86}$$

Thus, we precisely find

$$\gamma_k\widehat{Q}_k + Q - \Gamma\widehat{Q}_k = -(\Gamma\widehat{Q}_k - \gamma_k\widehat{Q}_k) + Q = -Q + Q = 0, \tag{87}$$

which completes the proof. □

As we have used the shorthand notation $\widehat{H}_k^5 := \widehat{(H_k^5)}_k$, we see that indeed

$$\gamma_k P_k + H_k^5 - \Gamma P_k = \gamma_k\widehat{H}_k^5 + H_k^5 - \Gamma\widehat{H}_k^5 = 0. \tag{88}$$

Returning to Equation (83), we find that it simplifies to

$$\begin{aligned}\dot{w}_k &= \gamma_k w_k - \beta_k w_k |w_k|^2 + \alpha(L_k^1(w) - L_k^2(w)) - \alpha^2[P_k||H^5](w) \\ &\quad + \mathscr{O}(|\alpha||w|^6 + |\alpha|^2|w|^5 + |\alpha|^3|w|^4),\end{aligned} \tag{89}$$

where we recall that L_k^1 and L_k^2 are defined as

$$\begin{aligned}&L_k^1(w) := [P_k||(\dots, \beta w_j|w_j|^2, \dots)](w) \text{ and} \\ &L_k^2(w) := [\beta_k w_k|w_k|^2||P](w),\end{aligned} \tag{90}$$

which are both polynomials of lower degree 4 or higher.

SN 2.4 The second reduction

Next, we wish to get rid of the term $\alpha(L_k^1(w) - L_k^2(w))$ in Equation (89). This follows along the same lines as in the previous reduction. We start by defining new variables

$$u_k = w_k - \alpha Q_k(w) \tag{91}$$

where each Q_k is a polynomial of lower degree 4 or higher. Note that by Lemma Supplementary Note 2:.18 we may write

$$w_k = u_k + \alpha Q_k(u) + \mathscr{O}(|\alpha|^2|u|^7)\,. \tag{92}$$

Using Equation (89) we obtain

$$\begin{aligned}\dot{u}_k &= \dot{w}_k - \alpha\frac{d}{dt}Q_k(w)\Bigg|\begin{array}{l}\dot{w}_j = \gamma_j w_j\\ \forall j\in\{1,\ldots,n\}\end{array} + \quad \mathscr{O}(|\alpha||w|^6)\\ &= \dot{w}_k - \alpha\Gamma Q_k(w) + \mathscr{O}(|\alpha||w|^6)\\ &= \gamma_k w_k - \beta_k w_k|w_k|^2 + \alpha(L_k^1(w) - L_k^2(w)) - \alpha^2[P_k||H^5](w) - \alpha\Gamma Q_k(w)\\ &+ \mathscr{O}(|\alpha||w|^6 + |\alpha|^2|w|^5 + |\alpha|^3|w|^4)\,.\end{aligned} \tag{93}$$

Next, substituting w_k by the right hand side of Equation (159) yields

$$\begin{aligned}\dot{u}_k &= \gamma_k w_k - \beta_k w_k|w_k|^2 + \alpha(L_k^1(w) - L_k^2(w)) - \alpha^2[P_k||H^5](w) - \alpha\Gamma Q_k(w)\\ &+ \mathscr{O}(|\alpha||w|^6 + |\alpha|^2|w|^5 + |\alpha|^3|w|^4)\\ &= \gamma_k u_k + \alpha\gamma_k Q_k(u) - \beta_k u_k|u_k|^2 + \alpha(L_k^1(u) - L_k^2(u)) - \alpha^2[P_k||H^5](u) - \alpha\Gamma Q_k(u)\\ &+ \mathscr{O}(|\alpha||u|^6 + |\alpha|^2|u|^5 + |\alpha|^3|u|^4)\\ &= \gamma_k u_k - \beta_k u_k|u_k|^2 + \alpha(L_k^1(u) - L_k^2(u) + \gamma_k Q_k(u) - \Gamma Q_k(u)) - \alpha^2[P_k||H^5](u)\\ &+ \mathscr{O}(|\alpha||u|^6 + |\alpha|^2|u|^5 + |\alpha|^3|u|^4)\,.\end{aligned} \tag{94}$$

It remains to choose Q_k such that

$$L_k^1(u) - L_k^2(u) + \gamma_k Q_k(u) - \Gamma Q_k(u) = 0\,. \tag{95}$$

Setting $S_k(u) := L_k^1(u) - L_k^2(u)$, Equation (95) becomes

$$\gamma_k Q_k + S_k - \Gamma Q_k = 0\,, \tag{96}$$

which is of the same form as Equation (84). It therefore follows from Lemma (Supplementary Note 2:.24) that a solution to Equation (95) is given by $Q_k = \widehat{S}_k := \widehat{(S_k)}_k$, if indeed this is well-defined. The following lemmas show that the non-resonance conditions of H_k^5 are enough to ensure $\widehat{S}_k$ exists.

Lemma Supplementary Note 2:.25. *The polynomial $S_k(u) = L_k^1(u) - L_k^2(u)$ may be expressed as the sum of terms $u_k^2\overline{R(u)}$ and $|u_j|^2R(u)$ for $j\in\{1,\ldots,n\}$ and with $R(u)$ a monomial term appearing in $P_k(u)$.*

Proof. We start with $L_k^2(u)$. By definition, we have

$$\begin{aligned}L_k^2(u) &= [\beta_k u_k|u_k|^2||P](u) = 2\beta_k u_k\overline{u}_k P_k(u) + \beta_k u_k^2\overline{P_k(u)}\\ &= 2\beta_k|u_k|^2P_k(u) + \beta_k u_k^2\overline{P_k(u)}\,.\end{aligned} \tag{97}$$

As $P_k(u)$ may be expressed as the sum of monomials that appear in $P_k(u)$ (tautologically), we see that $L_k^2(u)$ can indeed be written as the sum of terms $u_k^2\overline{R(u)}$ and $|u_j|^2 R(u)$, with $R(u)$ a monomial appearing in $P_k(u)$.

Next, recall that $L_k^1(u)$ is defined as

$$L_k^1(u) := [P_k||(\dots,\beta_j u_j|u_j|^2,\dots)](u)\,. \tag{98}$$

By definition of the bracket $[\bullet||\bullet]$, this means $L_k^1(u)$ is obtained from P_k by substituting terms u_j by $\beta_j u_j|u_j|^2$ and terms $\overline{u}_j$ by $\overline{\beta_j u_j|u_j|^2} = \overline{\beta_j u_j}|u_j|^2$. More precisely, if $R(u)$ is a monomial term of $P_k(u)$ given by

$$R(u) = u_1^{s_1}\dots u_n^{s_n}\overline{u}_1^{t_1}\dots\overline{u}_n^{t_n}\,, \tag{99}$$

then we find

$$\begin{aligned}
&[R(u)||(\dots,\beta_j u_j|u_j|^2,\dots)](u) \qquad (100)\\
=&\sum_{j=1}^n s_j u_1^{s_1}\dots u_j^{s_j-1}(\beta_j u_j|u_j|^2)\dots u_n^{s_n}\overline{u}_1^{t_1}\dots\overline{u}_n^{t_n} + \sum_{j=1}^n t_j u_1^{s_1}\dots u_n^{s_n}\overline{u}_1^{t_1}\dots\overline{u}_j^{t_j-1}\overline{(\beta_j u_j|u_j|^2)}\dots\overline{u}_n^{t_n}\\
=&\sum_{j=1}^n s_j\beta_j|u_j|^2 u_1^{s_1}\dots u_j^{s_j}\dots u_n^{s_n}\overline{u}_1^{t_1}\dots\overline{u}_n^{t_n} + \sum_{j=1}^n t_j\overline{\beta}_j|u_j|^2 u_1^{s_1}\dots u_n^{s_n}\overline{u}_1^{t_1}\dots\overline{u}_j^{t_j}\dots\overline{u}_n^{t_n}\\
=&\sum_{j=1}^n s_j\beta_j|u_j|^2 R(u) + \sum_{j=1}^n t_j\overline{\beta}_j|u_j|^2 R(u)\,.
\end{aligned}$$

Hence, by linearity of $[\bullet||\bullet]$ in the first slot (see Lemma Supplementary Note 2:.12), we see that $L_k^1(u)$ is again of the right form.

It follows that $S_k(u) = L_k^1(u) - L_k^2(u)$ can indeed be expressed as a sum of the given monomials terms. This completes the proof. □

Lemma Supplementary Note 2:.26. *Let $R(u)$ be a monomial and let $k,j \in \{1,\dots,n\}$ be fixed indices. The kth non-resonance condition of $R(u)$ is satisfied if and only if the kth non-resonance condition of $|u_j|^2R(u)$ is satisfied, if and only if the kth non-resonance condition of $u_k^2\overline{R(u)}$ is satisfied.*

Proof. We write

$$R(u) = u_1^{s_1}\dots u_n^{s_n}\overline{u}_1^{t_1}\dots\overline{u}_n^{t_n}\,, \tag{101}$$

so that the kth non-resonance condition of $R(u)$ is given by

$$s_1\omega_1 + \dots + s_n\omega_n - t_1\omega_1 - \dots - t_n\omega_n - \omega_k \neq 0\,. \tag{102}$$

It follows that the kth non-resonance condition of $|u_j|^2 R(u)$ is given by

$$\begin{aligned} \omega_j - \omega_j + & s_1\omega_1 + \dots + s_n\omega_n - t_1\omega_1 - \dots - t_n\omega_n - \omega_k \\ = & s_1\omega_1 + \dots + s_n\omega_n - t_1\omega_1 - \dots - t_n\omega_n - \omega_k \neq 0\,, \end{aligned} \tag{103}$$

which coincides with that of $R(u)$.
Likewise, the kth non-resonance condition of $u_k^2 \overline{R(u)}$ is given by

$$\begin{aligned} & 2\omega_k - s_1\omega_1 - \dots - s_n\omega_n + t_1\omega_1 + \dots + t_n\omega_n - \omega_k \\ = & -s_1\omega_1 - \dots - s_n\omega_n + t_1\omega_1 + \dots + t_n\omega_n + \omega_k \\ = & -(s_1\omega_1 + \dots + s_n\omega_n - t_1\omega_1 - \dots - t_n\omega_n - \omega_k) \neq 0\,, \end{aligned} \tag{104}$$

which is again equivalent to Equation (102). This completes the proof. □

Lemmas Supplementary Note 2:.25 and Supplementary Note 2:.26 guarantee that the kth non-resonance condition of $S_k(u) = L_k^1(u) - L_k^2(u)$ is satisfied if the kth non-resonance condition of $P_k = \widehat{H}_k^5$ is satisfied. As $\widehat{H}_k^5$ has the same monomial terms as H_k^5 (though rescaled), we see that the kth non-resonance conditions of $S_k(u)$ are indeed satisfied. Therefore, a solution to Equation (95) exists by Lemma (Supplementary Note 2:.24) and may be given by $Q_k = \widehat{S}_k$. Note that this choice of Q_k has lower degree 4 or higher, as we assumed throughout.

Returning to Equation (94), we finally arrive at

$$\dot{u}_k = \gamma_k u_k - \beta_k u_k |u_k|^2 - \alpha^2 [P_k||H^5](u) + \mathscr{O}(|\alpha||u|^6 + |\alpha|^2|u|^5 + |\alpha|^3|u|^4)\,. \tag{105}$$

We have therefore shown:

Proof of Theorem Supplementary Note 2:.4 and Proposition Supplementary Note 2:.9. The calculations in this section show that the successive coordinate transformations

$$\begin{aligned} w_k &= z_k - \alpha P_k(z) \\ u_k &= w_k - \alpha Q_k(w) \end{aligned} \tag{106}$$

bring the ODE

$$\dot{z}_k = \gamma_k z_k - \beta_k z_k |z_k|^2 + \alpha H_k(z)$$

into the form

$$\dot{u}_k = \gamma_k u_k - \beta_k u_k |u_k|^2 - \alpha^2 [P_k||H^5](u) + \mathscr{O}(|\alpha||u|^6 + |\alpha|^2|u|^5 + |\alpha|^3|u|^4)\,.$$

If we now set

$$G_k(u) := [P_k||H^5] = [\widehat{H}_k^5||H^5]\,,$$

then we indeed get

$$\dot{u}_k = \gamma_k u_k - \beta_k u_k |u_k|^2 - \alpha^2 G_k(u) + \mathscr{O}(|\alpha||u|^6 + |\alpha|^2|u|^5 + |\alpha|^3|u|^4)\,.$$

This completes the proof. □

Supplementary Note 3: Anomalous synchronization on a 4-node ring

Consider the four node ring network with a coupling function $h(z,w) = z\bar{w}$. leading to

$$\begin{aligned}
\dot{z}_1 &= \gamma_1 z_1 - \beta z_1|z_1|^2 + \alpha(z_1\overline{z}_2 + z_1\overline{z}_4) \\
\dot{z}_2 &= \gamma_2 z_2 - \beta z_2|z_2|^2 + \alpha(z_2\overline{z}_3 + z_2\overline{z}_1) \\
\dot{z}_3 &= \gamma_3 z_3 - \beta z_3|z_3|^2 + \alpha(z_3\overline{z}_4 + z_3\overline{z}_2) \\
\dot{z}_4 &= \gamma_4 z_4 - \beta z_4|z_4|^2 + \alpha(z_4\overline{z}_1 + z_4\overline{z}_3)
\end{aligned} \tag{107}$$

We set the parameters $\beta_k = -1$, $\gamma_k = \lambda + i\omega_k$, $\lambda = 1$ and $\omega_1 = 1 + \delta$, $\omega_2 = 1$ and $\omega_3 = 5$ and $\omega_4 = 6$ for performing the simulation of Eq. (107). We then vary the mismatch δ and coupling α. Notice that by a naive inspection of the original equations we obtain

$$\dot{\theta}_k = \omega_k + \alpha \sum_{\ell=1}^{4} A_{k\ell} \sin\theta_\ell$$

thus, instead of a diffusive interaction we would obtain a interaction akin to forcing [2]. For each set of α and δ values, we simulate the network for 50000s with 0.01 time step. We remove first 10000s from as transient and compute the unwrapped phases. As we are interested in the phase synchronization, we introduce a new variable for the phase differences, $\phi = \theta_1 - \theta_2$, and a naive calculation leads to

$$\dot{\phi} = \delta + \alpha[\sin\theta_2 + \sin\theta_4 - \sin\theta_1 - \sin\theta_3] + O(\alpha^2).$$

Because $\omega_2, \omega_3 \ll \omega_1$, we can average over the fast oscillations and neglect contributions from these phases then $\theta_2 = \theta_1 + \phi$ we would obtain a interaction term as $\alpha[\sin\theta_1\cos\phi + \cos\theta_1\sin\phi] + O(\alpha^2)$, however, since θ_1 is a fast variable for ϕ we also average over θ_1. Thus the whole interaction term linear in α vanishes.

We calculate the mean synchronization error as

$$E = \frac{1}{T}\sum_{t=1}^{T} |\phi(t)|. \tag{108}$$

The synchronization error for varying $\delta = -0.2$ to 0.2 with 0.01 step size and $\alpha = 0.0$ to 0.5 with step size 0.025, we observed a synchronization tongue scales with $\alpha \propto \sqrt{\delta}$ (Supplementary Fig. 3).

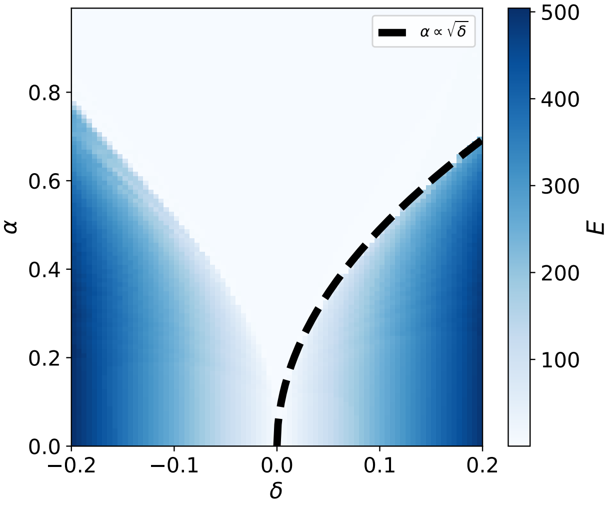

Supplementary Figure 3: **Arnold tongue represents the phase synchronization between nodes 1 and 2**. The tongue is illustrated for varying mismatch δ and coupling strength α. As the absolute value of $|\delta| \ll 1$, synchronization occurs for small coupling strength. However, if the mismatch is high then also stronger coupling is needed to emerge synchronization. The borderline between synchrony and asynchrony scales with $\alpha \propto \sqrt{\delta}$.

SN 3.1 Explanation of anomalous synchronization via normal form theory

Recall that we may bring this ODE into the form

$$\begin{aligned}
\dot{u}_1 &= \gamma_1 u_1 - \beta u_1 |u_1|^2 \\
&\quad - \alpha^2 u_1 \left(\frac{\overline{u}_2 \overline{u}_4}{\overline{\gamma}_2} + \frac{\overline{u}_4 \overline{u}_2}{\overline{\gamma}_4} + \frac{\overline{u}_2 \overline{u}_2}{\overline{\gamma}_2} + \frac{\overline{u}_4 \overline{u}_4}{\overline{\gamma}_4} + \frac{\overline{u}_2 u_3}{\overline{\gamma}_2} + \frac{\overline{u}_2 u_1}{\overline{\gamma}_2} + \frac{\overline{u}_4 u_3}{\overline{\gamma}_4} + \frac{\overline{u}_4 u_1}{\overline{\gamma}_4} \right) + \text{h.o.t.} \\
\dot{u}_2 &= \gamma_2 u_2 - \beta u_2 |u_2|^2 \\
&\quad - \alpha^2 u_2 \left(\frac{\overline{u}_1 \overline{u}_3}{\overline{\gamma}_1} + \frac{\overline{u}_3 \overline{u}_1}{\overline{\gamma}_3} + \frac{\overline{u}_1 \overline{u}_1}{\overline{\gamma}_1} + \frac{\overline{u}_3 \overline{u}_3}{\overline{\gamma}_3} + \frac{\overline{u}_3 u_4}{\overline{\gamma}_3} + \frac{\overline{u}_3 u_2}{\overline{\gamma}_3} + \frac{\overline{u}_1 u_4}{\overline{\gamma}_1} + \frac{\overline{u}_1 u_2}{\overline{\gamma}_1} \right) + \text{h.o.t.} \\
\dot{u}_3 &= \gamma_3 u_3 - \beta u_3 |u_3|^2 \\
&\quad - \alpha^2 u_3 \left(\frac{\overline{u}_2 \overline{u}_4}{\overline{\gamma}_2} + \frac{\overline{u}_4 \overline{u}_2}{\overline{\gamma}_4} + \frac{\overline{u}_2 \overline{u}_2}{\overline{\gamma}_2} + \frac{\overline{u}_4 \overline{u}_4}{\overline{\gamma}_4} + \frac{\overline{u}_2 u_3}{\overline{\gamma}_2} + \frac{\overline{u}_2 u_1}{\overline{\gamma}_2} + \frac{\overline{u}_4 u_3}{\overline{\gamma}_4} + \frac{\overline{u}_4 u_1}{\overline{\gamma}_4} \right) + \text{h.o.t.} \\
\dot{u}_4 &= \gamma_4 u_4 - \beta u_4 |u_4|^2 \\
&\quad - \alpha^2 u_4 \left(\frac{\overline{u}_1 \overline{u}_3}{\overline{\gamma}_1} + \frac{\overline{u}_3 \overline{u}_1}{\overline{\gamma}_3} + \frac{\overline{u}_1 \overline{u}_1}{\overline{\gamma}_1} + \frac{\overline{u}_3 \overline{u}_3}{\overline{\gamma}_3} + \frac{\overline{u}_3 u_4}{\overline{\gamma}_3} + \frac{\overline{u}_3 u_2}{\overline{\gamma}_3} + \frac{\overline{u}_1 u_4}{\overline{\gamma}_1} + \frac{\overline{u}_1 u_2}{\overline{\gamma}_1} \right) + \text{h.o.t.}
\end{aligned} \tag{109}$$

We will assume that $\gamma_1 \approx \gamma_2$. In particular, we consider the possibility that $\gamma_1 = \gamma_2$. In that case we have $\gamma_1 + \overline{\gamma_2} = 2\,\mathrm{Re}(\gamma_1)$, which may be arbitrarily small. Therefore, we may not assume that

$\gamma_1 + \overline{\gamma_2} \neq 0$. Other than this, there are no relevant restrictions. I.e., $\gamma_1, \ldots, \gamma_4, \gamma_2 - \overline{\gamma_3} \ldots \gamma_3 - \overline{\gamma_4}$ are all sufficiently large. It follows that we may bring equation (109) into the form

$$\begin{aligned}
\dot{v}_1 &= \gamma_1 v_1 - \beta v_1 |v_1|^2 - \epsilon \frac{v_1 \overline{v}_2 v_1}{\overline{\gamma}_2} + \mathscr{O}(|\epsilon, v|^5) \\
\dot{v}_2 &= \gamma_2 v_2 - \beta v_2 |v_2|^2 - \epsilon \frac{v_2 \overline{v}_1 v_2}{\overline{\gamma}_1} + \mathscr{O}(|\epsilon, v|^5) \\
\dot{v}_3 &= \gamma_3 v_3 - \beta v_3 |v_3|^2 - \epsilon \frac{v_3 \overline{v}_2 v_1}{\overline{\gamma}_2} + \mathscr{O}(|\epsilon, v|^5) \\
\dot{v}_4 &= \gamma_4 v_4 - \beta v_4 |v_4|^2 - \epsilon \frac{v_4 \overline{v}_1 v_2}{\overline{\gamma}_1} + \mathscr{O}(|\epsilon, v|^5) \,,
\end{aligned} \tag{110}$$

where $\epsilon = \alpha^2$. Note that the network topology has changed drastically. Moreover, performing a phase reduction and introducing the phase difference $\psi = \theta_1 - \theta_2$ we obtain

$$\dot{\psi} = \delta - c\alpha^2 \sin\psi$$

where c is a constant depending on γ_1. By analyzing the fixed points of this equation we obtain the synchronization tongue behavior where the critical coupling α_c for synchronization scales as $\sqrt{\delta}$.

Supplementary Note 4: Phase reduction for $h = (z^2 + z)\bar{w}$ and resonance $\omega_1 - \omega_{2,4} + \omega_3 = 0$

For simplicity we fix $\beta_k = -1$ and obtain $\gamma_k = r_0^2 + i\omega_k$. We also introduce $\Delta_{pq} = \omega_p - \omega_q$. Note that $r(t) = r_0 + h.o.t$ along with $\bar{z}\dot{z} = ir_0^2\dot{\theta} + h.o.t.$ and

$$\frac{1}{\gamma_p + \bar{\gamma}_q} = \frac{2r_0^2 - i\Delta_{pq}}{4r_0^4 + \Delta_{pq}^2} \tag{111}$$

Replacing these observations into Eq. (6) of the main manuscript and performing the reduction we obtain the functions in Eq. (7-8) of the main manuscript as

$$\begin{aligned}\rho_{pq}(\phi) &= -\frac{\Delta_{pq}}{4r_0^4 + \Delta_{pq}^2}\cos\phi + \frac{2r_0^2}{4r_0^4 + \Delta_{pq}^2}\sin\phi \\ \sigma_{pqr}(\phi) &= -\chi_{pqr}\sin\phi + \upsilon_{pqr}\cos\phi\end{aligned} \tag{112}$$

where

$$\chi_{pqr} = r_0^2\left(\frac{4}{4r_0^4 + \Delta_{pq}^2} + \frac{4}{4r_0^4 + \Delta_{pr}^2} + \frac{1}{r_0^4 + \omega_q^2} + \frac{1}{r_0^4 + \omega_r^2}\right)$$

and

$$\upsilon_{pqr} = -\frac{2\Delta_{pq}}{4r_0^4 + \Delta_{pq}^2} - \frac{2\Delta_{pr}}{4r_0^4 + \Delta_{pr}^2} + \frac{\omega_q}{r_0^4 + \omega_q^2} + \frac{\omega_r}{r_0^4 + \omega_r^2}$$

Supplementary Note 5: Slow phase dynamics for $h = (z^2 + z)\bar{w}$ and resonance $\omega_1 - \omega_{2,4} + \omega_3 = 0$

We consider networks of n coupled oscillators

$$\dot{z}_k = f_k(z_k) + \alpha\sum_{\ell=1}^{n} A_{k\ell}h_k(z_k, z_\ell) \tag{113}$$

where $z_k \in \mathbb{C}$ is the state of the kth oscillator, $f_k : \mathbb{C} \to \mathbb{C}$ is its isolated vector field, $h_k : \mathbb{C}\times\mathbb{C} \to \mathbb{C}$ is the pairwise coupling function, $\boldsymbol{A} = (A_{ij})_{i,j=1}^n$ is the adjacency matrix describing the network structure, and $\alpha > 0$ is the coupling strength. We then generate a multivariate time series for a four-node ring network, as illustrated in Figure 4 (a), with nonlinear pairwise coupling function

$$h(z, w) = (z + z^2)\bar{w}. \tag{114}$$

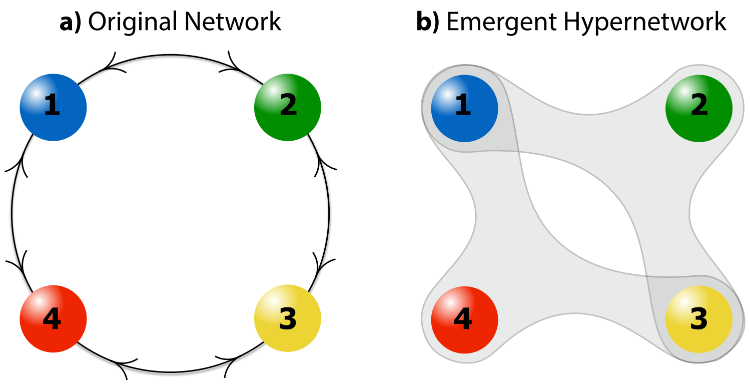

Supplementary Figure 4: **Emergent hypernetworks with triplet interactions.** (a) The original ring network. (b) Hypernetwork recovered from original dynamics data. The state of a given node is influenced by triplet interactions. We show that as along as some sparsity is imposed when the model is obtained from data, only hypernetworks can be found. Our theory predicts the emergence of the hypernetwork determined by the original coupling function h, the network, and the resonance relations among the isolated frequencies.

We fix $\lambda = 0.15$, $\omega_1 = 1.01$, $\omega_2 = 2.5$, $\omega_3 = 1.5$, $\omega_4 = 2.49$, and $\alpha = 0.18$. Numerical integration of complex differential equations is used to solve the differential equations for 10000-sec with 0.01-sec time-step. We discard the first 5000-sec points as transient, and we obtain a multivariate time series $\{z_1(t), z_2(t), z_3(t), z_4(t)\}_{t=1}^{5000}$.

Next, we aim at obtaining a model from the multivariate time series of z. Because α is small and the isolated orbit is exponentially stable, the amplitude of each time series is slightly affected by $|z_k| \approx \sqrt{\lambda} + O(\alpha)$ as illustrated in Figure. 5 (a), and the dynamics is captured by the phases $\theta_k(t)$ of $z_k(t)$. Therefore, we perform a polar decomposition $z_k(t) = r_k(t)e^{i\theta_k(t)}$ to get the unwrapped phase of each time series and obtain governing equations of the model from its phase dynamics.

Each phase θ_k has a frequency close to ω_k, as illustrated in Figure 5 (b). This means that the growth of the phases is almost linear with coupling terms as perturbations. In fact, the coupling terms generically contain fast variables such as phases θ's and slow variables involving the resonant combinations of phases such as $\theta_1 - \theta_2 + \theta_3$ that change slowly in time. Therefore, we subtract the linear growth of the phases to analyse the effects of the coupling. To this end, we introduce

$$\vartheta_k(t) = \theta_k(t) - \Omega_k t, \tag{115}$$

where Ω_k is obtained from data under the resonance condition $\Omega_1 - \Omega_i + \Omega_3 = 0$, with $i = 2, 4$. In the new phases the coupling has the same magnitude as the frequency mismatch $\omega_1 - \omega_i + \omega_3$, with $i = 2, 4$. Finally, we obtain a model for ϑ_k. We assume the model

$$\dot{\vartheta}_k = \varepsilon_k + H_k(\vartheta_1, \vartheta_2, \vartheta_3, \vartheta_4)$$

where $H_k = \sum[c_p^k \sin\vartheta_p + d_p^k \cos\vartheta_p] + \sum[c_{p,q}^k \sin(\vartheta_p - \vartheta_q) + d_{p,q}^k \cos(\vartheta_p - \vartheta_q] + \sum[c_{pq}^k \sin(\vartheta_p + \vartheta_q - \vartheta_k) + d_{pq}^k \cos(\vartheta_p + \vartheta_q - \vartheta_k)]$. Note that this includes pairwise and triplet interactions. We solve

for the coefficients to obtain the least square approximation and we impose sparsity by eliminating coefficients below a threshold $\tau = 10^{-4}$. The technique is discussed along with the package to perform the recovery as discussed in the main text. The model recovery yields

$$\begin{aligned} \dot{\vartheta}_{1,3} &= \varepsilon_{1,3} + r_{1,3}(\vartheta_1, \vartheta_2, \vartheta_3) + s_{1,3}(\vartheta_1, \vartheta_4, \vartheta_3) \\ \dot{\vartheta}_{2,4} &= \varepsilon_{2,4} + r_{2,4}(\vartheta_1, \vartheta_{2,4}, \vartheta_3) \end{aligned} \tag{116}$$

where s and r correspond to triplets in H_k with nonzero coefficients.

At first sight, the model recovery with triplets is remarkable because the original equations have only pairwise interactions. Nonetheless, a hypernetwork describes the data Figure 4 (b). We show that when $\lambda \ll 1$ and $\alpha \ll 1$ recovering a hypernetwork from data is not a coincidence. As long as the coupling h is nonlinear, by measuring the original variables of Eq. (113) and performing a sparse model recovery only hypernetworks can be found as they are normal forms of the original equations.

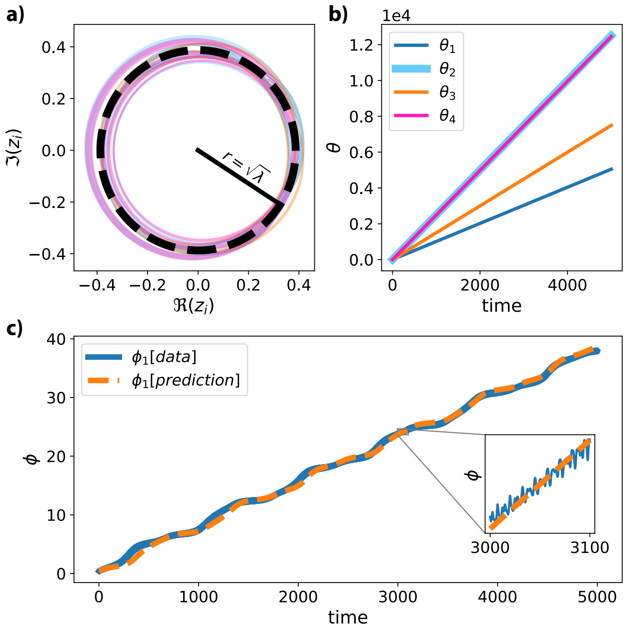

Supplementary Figure 5: **Time series and emergent hypernetwork prediction.** The simulation was performed on a ring network (see Supplementary Fig. 4 (a)). (a) Amplitudes (solid lines) are slightly affected by the coupling and remaining close to a circle with radius $r = \sqrt{\lambda}$ (dashed circle). (b) Unwrapped phases θ_i growth. (c) Time series of the slow phase ϕ_1 from data (solid) and the prediction of the emergent hypernetwork (dashed) capturing higher-order interactions (see Supplementary Fig. 4 (b)).

SN 5.1 Emergent hypernetwork predicts data behaviour

To illustrate prediction capabilities of emergent hypernetworks, we introduce the slow phases

$$\begin{aligned}\phi_1 &= \theta_1 - \theta_2 + \theta_3, \\ \phi_2 &= \theta_1 - \theta_4 + \theta_3,\end{aligned} \tag{117}$$

where the coupling strength is comparable to the frequency mismatch $\omega_1 - \omega_2 + \omega_3$. Our normal form theory predicts the emergent hypernetwork phase dynamics described in Eq. (15). We obtain the vector fields of the coupled slow phases ϕ_1 and ϕ_2 analytically as described in the section above.

Next, we simulate the vector fields obtained from first principles using an adaptative Runge-Kutta method of 4th order. We treat the initial condition as unknown and perform a optimization to obtain the the initial condition that provides the minimum least square error between the data of the slow phase and the simulations and theory. In Figure 5 c), we compare our predictions and slow phases estimated from data. The theoretical prediction is in excellent agreement with the data with an error in the prediction of less than 5% per cycle of the slow phase.

Supplementary Note 6: Model recovery of a 3-path with coupling $h = (z^2 + z)\bar{w}$ and resonance $\omega_1 - \omega_2 + \omega_3 = 0$

Now we consider the model for 3-nodes on a chain (Supplementary Fig. 6) that reads as We consider the network ODE

$$\begin{aligned}\dot{z}_1 &= \gamma_1 z_1 - \beta z_1 |z_1|^2 + \alpha(z_1\overline{z_2} + z_1^2\overline{z_2}) \\ \dot{z}_2 &= \gamma_2 z_2 - \beta z_2 |z_2|^2 + \alpha([z_2\overline{z_1} + z_2^2\overline{z_1}] + [z_2\overline{z_3} + z_2^2\overline{z_3}]) \\ \dot{z}_3 &= \gamma_3 z_3 - \beta z_3 |z_3|^2 + \alpha(z_3\overline{z_2} + z_3^2\overline{z_2}),\end{aligned} \tag{118}$$

To integrate Eq. (118) of the main manuscript for 3-node chain with $\omega_1 = 1.01$, $\omega_2 = 2.5$ and $\omega_3 = 1.5$, we employed a wrapper of ODEPACK routine. Numerical integration for $\alpha = 0.18$ and $\delta = 0.01$ was performed for 10000s with 0.01 time step. We discard the first 5000s points as transient. Using the simulated phases θ_i, we introduce new phases in Eq. (3) of the main manuscript where $\Omega_1 = 1.0$, $\Omega_2 = 2.5$, $\Omega_3 = 1.5$. Applying the sequential thresholded least-squares method on these new phases ϑ_i with thresholding parameter $\lambda = 10^{-4}$ we obtain

$$\dot{\vartheta}_1 = 0.01 - 0.001\cos(\vartheta_1 - \vartheta_2 + \vartheta_3) \tag{119}$$

$$\dot{\vartheta}_2 = -0.001 + 0.005\cos(\vartheta_1 - \vartheta_2 + \vartheta_3) \tag{120}$$

$$\dot{\vartheta}_3 = -0.001 - 0.001\cos(\vartheta_1 - \vartheta_2 + \vartheta_3) \tag{121}$$

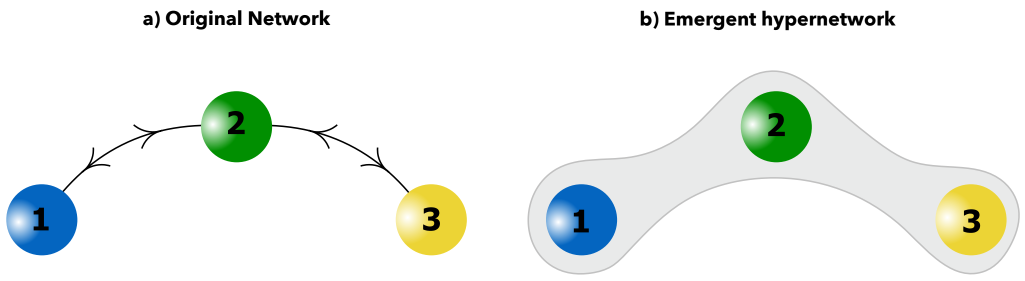

Supplementary Figure 6: **Emergent hypernetworks with triplet interaction.** Inset a) shows the original chain network. Each isolated node dynamics is close to a Hopf bifurcation. The pairwise coupling function h is nonlinear given with $h = (z^2 + \bar{z})w$. Inset b) shows the hypernetwork learnt from the phase dynamics data of the original dynamics. The state of a given node is influenced by the triple interaction of its own state in combination of the incoming links. Our theory also predicts the emergent of such hypernetwork. The hypernetwork emerges as a combination of the original coupling function h, the network, and the resonance relations of the isolated frequencies.

Because the norms of the functions r_k and s_k are small, we introduce the slow phases

$$\phi \quad = \quad \theta_1 - \theta_2 + \theta_3$$

We then also perform a reconstruction for the slow phases ϕ using the same method and obtain

$$\dot{\phi} = 0.010 + 0.001 \sin(\phi) - 0.006 \cos(\phi) \tag{122}$$

We show the model prediction and data for the slow phase in Figure 7.

SN 6.1 Emergent network explanation

The normal-form for this system is given by

$$\begin{aligned}
\dot{u}_1 &= \gamma_1 u_1 - \beta u_1 |u_1|^2 - \alpha^2 \left(\frac{1}{\gamma_1 + \overline{\gamma}_2} \right) u_1^2 \overline{u}_2 u_3 \\
\dot{u}_2 &= \gamma_2 u_2 - \beta u_2 |u_2|^2 - \alpha^2 \left(\frac{2}{\gamma_2 + \overline{\gamma}_3} + \frac{2}{\gamma_2 + \overline{\gamma}_1} + \frac{1}{\overline{\gamma}_3} + \frac{1}{\overline{\gamma}_1} \right) u_2^2 \overline{u}_1 \overline{u}_3 \\
\dot{u}_3 &= \gamma_3 u_3 - \beta u_3 |u_3|^2 - \alpha^2 \left(\frac{1}{\gamma_3 + \overline{\gamma}_2} \right) u_3^2 \overline{u}_2 u_1 \,.
\end{aligned} \tag{123}$$

The interaction now becomes forth order in u. A phase reduction leads to the triplet interaction recovered numerically.

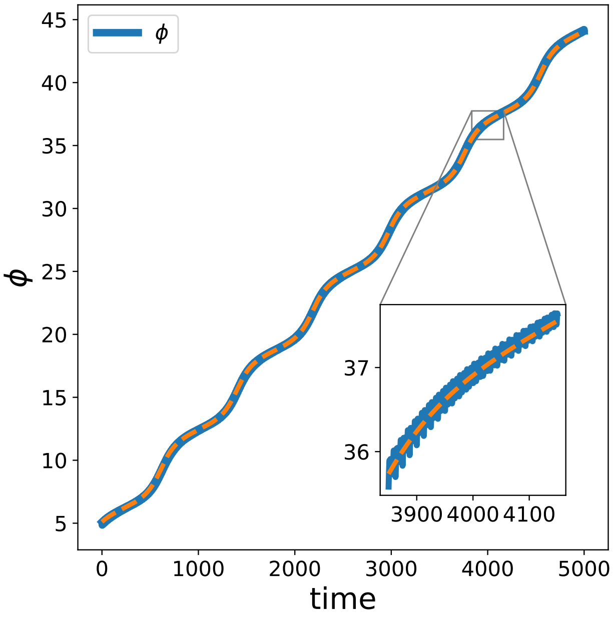

Supplementary Figure 7: New slow phase variable ϕ (blue curve) were computed from the data collected from the simulations of Eq. (1) on a path. Then Eq. 150 (orange dashed curve) reconstructed from data of ϕ using sequential thresholded least squares method.

Supplementary Note 7: 6 nodes network examples with $h(z, w) = z\bar{w}$

We consider the network presented in Figure 8a) with the coupling $h(z, w) = z\bar{w}$ leading to

$$\begin{aligned}
\dot{z}_1 &= \gamma_1 z_1 - \beta z_1|z_1|^2 + \alpha(z_1\overline{z}_2 + z_1\overline{z}_3 + z_1\overline{z}_4) \\
\dot{z}_2 &= \gamma_2 z_2 - \beta z_2|z_2|^2 + \alpha(z_2\overline{z}_1 + z_2\overline{z}_3 + z_2\overline{z}_5) \\
\dot{z}_3 &= \gamma_3 z_3 - \beta z_3|z_3|^2 + \alpha(z_3\overline{z}_1 + z_3\overline{z}_2 + z_3\overline{z}_6) \\
\dot{z}_4 &= \gamma_4 z_4 - \beta z_4|z_4|^2 + \alpha(z_4\overline{z}_5 + z_4\overline{z}_6 + z_4\overline{z}_1) \\
\dot{z}_5 &= \gamma_5 z_5 - \beta z_5|z_5|^2 + \alpha(z_5\overline{z}_4 + z_5\overline{z}_6 + z_5\overline{z}_2) \\
\dot{z}_6 &= \gamma_6 z_6 - \beta z_6|z_6|^2 + \alpha(z_6\overline{z}_4 + z_6\overline{z}_5 + z_6\overline{z}_3)\,.
\end{aligned} \tag{124}$$

We will assume either one of

1. $\gamma_2 \approx \gamma_5$, $\gamma_1 \approx \gamma_6$ and $\gamma_3 \approx \gamma_4$, with $\gamma_1 \not\approx \gamma_2$ (and hence $\gamma_1, \gamma_6 \not\approx \gamma_2, \gamma_5$), $\gamma_2 \not\approx \gamma_3$ and $\gamma_1 \not\approx \gamma_3$.
2. $\gamma_2 \approx \gamma_5$ and $\gamma_i \not\approx \gamma_j$ for all $i, j \in \{1, \ldots, 6\}$ with $i \neq j$ and $(i, j) \neq (2, 5), (5, 2)$.

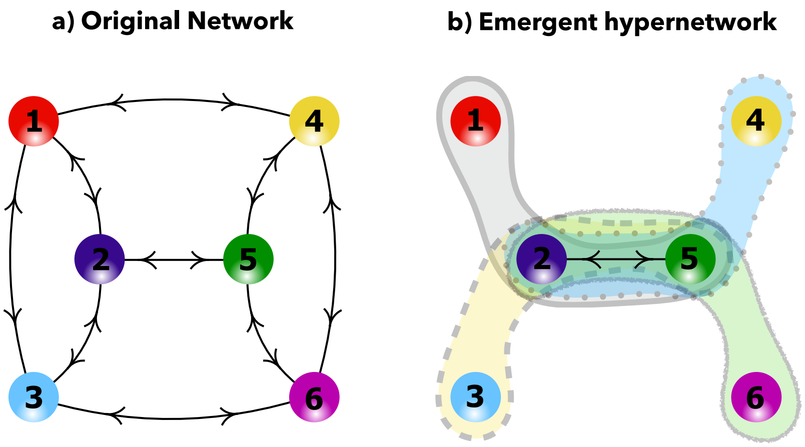

Supplementary Figure 8: **Emergent hypernetworks with triplet interaction.** Inset a) shows the original network. Each isolated node dynamics is close to a Hopf bifurcation. The pairwise coupling function $h(z, w) = z\bar{w}$. Inset b) shows the hypernetwork reconstructed from the phase dynamics of the original dynamics. The state of a given node is influenced by the triple interaction of its own state in combination of the incoming links. Our theory also predicts the emergent of such hypernetwork. The hypernetwork emerges as a combination of the original coupling function $h(z, w) = z\bar{w}.$, the network, and the resonance relations of the isolated frequencies.

In each case, we may transform ODE (124) into

$$\begin{aligned}
\dot{v}_1 &= \gamma_1 v_1 - \beta v_1 |v_1|^2 - \epsilon \frac{v_1 \overline{v}_2 v_5}{\overline{\gamma}_2} + \mathscr{O}(|\epsilon, v|^5) \\
\dot{v}_2 &= \gamma_2 v_2 - \beta v_2 |v_2|^2 - \epsilon \frac{v_2 \overline{v}_5 v_2}{\overline{\gamma}_5} + \mathscr{O}(|\epsilon, v|^5) \\
\dot{v}_3 &= \gamma_3 v_3 - \beta v_3 |v_3|^2 - \epsilon \frac{v_3 \overline{v}_2 v_5}{\overline{\gamma}_2} + \mathscr{O}(|\epsilon, v|^5) \\
\dot{v}_4 &= \gamma_4 v_4 - \beta v_4 |v_4|^2 - \epsilon \frac{v_4 \overline{v}_5 v_2}{\overline{\gamma}_5} + \mathscr{O}(|\epsilon, v|^5) \\
\dot{v}_5 &= \gamma_5 v_5 - \beta v_5 |v_5|^2 - \epsilon \frac{v_5 \overline{v}_2 v_5}{\overline{\gamma}_2} + \mathscr{O}(|\epsilon, v|^5) \\
\dot{v}_6 &= \gamma_6 v_6 - \beta v_6 |v_6|^2 - \epsilon \frac{v_6 \overline{v}_5 v_2}{\overline{\gamma}_5} + \mathscr{O}(|\epsilon, v|^5) \,,
\end{aligned} \tag{125}$$

where $\epsilon = \alpha^2$ leading to the emergent higher order network displayed in Figure 8b).

Supplementary Note 8: Model recovery and normal form representation

Let $x \in \mathbb{R}^m$ and consider

$$\dot{x} = F(x) \tag{126}$$

We assume for simplicity that $F : \mathbb{R}^m \to \mathbb{R}^m$ is a polynomial map and

$$\dot{x}_i = \sum_{j=1}^{k} a_{ij} p_j(x), \tag{127}$$

where x_i is the ith coordinate of x and p_j's form a basis of homogeneous polynomials. Notice that in a network context, $x \in \mathbb{R}^m$ would represent the state vector of the network and F would model isolated dynamics and interactions. Once a trajectory $x(t)$ and $\dot{x}(t)$ are known, we perform a model recovery as follows. Fix a sampling h and introduce

$$V = \begin{pmatrix} \dot{x}_1(0) & \dot{x}_2(0) & \dots & \dot{x}_m(0) \\ \dot{x}_1(h) & \dot{x}_2(h) & \dots & \dot{x}_m(h) \\ \vdots & \vdots & \ddots & \vdots \\ \dot{x}_1(T) & \dot{x}_2(T) & \dots & \dot{x}_m(T) \end{pmatrix} \quad \text{and} \quad X = \begin{pmatrix} x_1(0) & x_2(0) & \dots & x_m(0) \\ x_1(h) & x_2(h) & \dots & x_m(h) \\ \vdots & \vdots & \ddots & \vdots \\ x_1(T) & x_2(T) & \dots & x_m(T) \end{pmatrix}$$

along with

$$\Phi(X) = \begin{pmatrix} p_1(x(0)) & p_2(x(0)) & \cdots & p_k(x(0)) \\ p_1(x(h)) & p_2(x(h)) & \cdots & p_k(x(h)) \\ \vdots & \vdots & \ddots & \vdots \\ p_1(x(T)) & p_2(x(T)) & \cdots & p_k(x(T)) \end{pmatrix}$$

Let v_i be the ith column of V and $\xi_i = (a_{i1}, a_{i2}, \dots, a_{ik})^*$. Here, * denotes the transpose. Then by construction

$$\Phi(X)\xi_i = v_i \tag{128}$$

and if for large T the operator Φ is full rank the solution of Eq. (128) is unique. Solving this equation for all coordinates, we recover the differential equation.

In data, however, due to numerical round-off errors or noise Eq. (128) is perturbed and one seeks for solutions allowing a small error $\|\Phi(X)\xi_i - v_i\| < \varepsilon_0$ but under a model simplification such as imposing that some coefficients of ξ_i are zero, that is, looking for sparse solutions.

The sparse model recovery of the coefficients ξ_i is the problem

$$\min_{q\in\mathbb{R}^k} \|q\|_0 \quad \text{subjected to} \quad \|\Phi(X)q - v_i\| \le \lambda$$

for a suitably chosen $\lambda > 0$.

Now we are ready to prove the following

Theorem Supplementary Note 8:.1. *Consider Eq. (127) and the following assumptions*

(H0) Eq. (127) is generic (coefficients a_{ij} are non vanishing)

(H1) Eq (127) has a normal form

$$\dot{y} \;=\; G(y) + R(y) \tag{129}$$

where G contains no non-resonant terms and $\|R(y)\| = O(y^{d+1})$ for some large d. Moreover the coordinates of y have the expansion

$$\dot{y}_i = \sum_{j=1}^{k} b_{ij} p_j(y) + R_i(y)$$

(H2) The trajectories $\{x(t)\}_{t=0}^{T}$ as well as $\{\dot{x}(t)\}_{t=0}^{T}$ are given with T sufficiently large and stay in a sufficiently small neighbourhood V_ε of the origin such that

$$\sup_{t\in[0,T]} \|x(t)\|_{C^1} \le \varepsilon$$

for initial conditions in an open neighbourhood of the origin.

(H3) The operator Φ is full rank.

Then there exist $\lambda = \lambda(\varepsilon, d) > 0$ such the solution to the sparse recovery problem

$$\min_{q\in\mathbb{R}^k} \|q\|_0 \quad \textit{subjected to} \quad \|\Phi(X)q - v_i\|_2 \le \lambda$$

is the vector of coefficients of $(b_{i1}, b_{i2}, \ldots, b_{ik})^*$ of the normal form of Eq. (127)

Proof. We break the arguments into three steps:

Step1: Approximations and Uniqueness solutions. By normal form theory there are functions Q_1 and Q_2 such that

$$x = y + Q_1(y) \quad \text{and} \quad y = x + Q_2(x) \tag{130}$$

where $\|Q_1(y)\| = O(\|y\|^2)$ and $\|Q_2(x)\| = O(\|x\|^2)$. Given a trajectory $y(t)$ we construct the matrix Y in the same manner as X and consider

$$u_i = \begin{pmatrix} \dot{y}_i(0) \\ \dot{y}_i(h) \\ \vdots \\ \dot{y}_i(T) \end{pmatrix} \quad \text{and} \quad \rho_i(Y) = \begin{pmatrix} R_i(y(0)) \\ R_i(y(h)) \\ \vdots \\ R_i(y(T)) \end{pmatrix}$$

As the basis is formed by homogeneous polynomials, using Eq. (130) we conclude that there is L such that

$$\|\Phi(X) - \Phi(Y)\|_2 \leq L\varepsilon^2$$

By $(H3)$ $\Phi(X)$ is full rank and for ε^2 small enough, we conclude that $\Phi(Y)$ is also full rank since the rank is lower semicontinuous. Next notice that the equation

$$\Phi(Y)\zeta_i + \rho_i(Y) = u_i \tag{131}$$

also has a solution $\zeta_i = (b_{i1}, b_{i2}, \ldots, b_{ik})^*$ by construction and it is unique since $\Phi(Y)$ is full rank. Furthermore, in V_ε there is a constant M such that

$$\|\rho_i(Y)\|_2 \leq M\varepsilon^2, \quad \forall i \in \{1, \ldots, m\}$$

Using Eq. (130) we obtain

$$v_i = u_i + z_i \tag{132}$$

where z_i corresponds to terms as $DP(y)\dot{y}$. By $(H2)$ trajectories stay in the neighbourhood V_ε, thus, there is a constant C such that

$$\|z_i\|_2 \leq C\varepsilon^2, \quad \forall i \in \{1, \ldots, m\}$$

Step 2: A sparse solution. Consider the unique solution ζ_i of Eq. (131) and let $\sigma_i = \|\zeta_i\|_0$. Consider the set

$$B_{\lambda,\sigma_i} = \{q \in \mathbb{R}^k : \|q\|_0 \leq \sigma_i \text{ and } \|\Phi(X)q - v_i\|_2 \leq \lambda\}$$

Now we claim that if $\lambda := (L\|\zeta_i\|_2 + C + M)\varepsilon^d$ then $\zeta_i \in B_{\lambda,\sigma_i}$. Indeed, consider

$$\begin{aligned} \|\Phi(X)\zeta_i - v_i\|_2 &= \|\Phi(X)\zeta_i + \Phi(Y)\zeta_i - \Phi(Y)\zeta_i - v_i\|_2 \\ &= \|[\Phi(X) - \Phi(Y)]\zeta_i + \Phi(Y)\zeta_i - u_i - z_i + \rho_i - \rho_i\|_2 \\ &= \|[\Phi(X) - \Phi(Y)]\zeta_i - z_i - \rho_i\|_2 \\ &\leq \|\Phi(X) - \Phi(Y)\|_2\|\zeta_i\|_2 + \|z_i\|_2 + \|\rho_i\|_2 \\ &\leq (L\|\zeta_i\|_2 + M + C)\varepsilon^2 \end{aligned}$$

Step 3: Uniqueness. Assume that there is $\eta \in B_{\lambda,\sigma_i}$ with $\|\eta\|_0 < \sigma_i$. Since $\Phi(X)$ is full rank, this implies that there is $\hat{R}$ such that $\|\hat{R}(x)\|_2 \leq K\|x\|_2^{d+1}$ for some K and $\dot{x} = \hat{G}(x) + \hat{R}(x)$. Thus, $\hat{G}$ has fewer coefficients than G, implying that either G must have a non-resonant term or $(H0)$ was violated. This contracts $(H1)$ and completes the proof.

□

Remark Supplementary Note 8:.2. Assumption $H2$ is natural in our context. Notice since the isolated system has a limit cycle near the origin. Thus, an open set of initial conditions is attracted to the cycles and stays for all times near the origin where we control the norm of solutions [3]. When coupling such dynamics to a network this behaviour persists.

Remark Supplementary Note 8:.3. Assumption $H3$ is in general not restrictive. If solutions of $\dot{x} = F(x)$ are not degenerated such as all solutions converge to fixed points, then typically $\Phi(X)$ is full rank. In fact, if solutions converge to an attractor, we can adapt the basis to the dynamics such that in the adapted basis $\Phi^*(X)\Phi(X)$ is close to identity for large T [4]. This implies that $\Phi(X)$ is close to orthogonal.

Remark Supplementary Note 8:.4. Another interesting case is when

$$\dot{x} = F(x) + U(t, x)$$

and U has fast oscillations. This happens typically in phase dynamics when we subtract the trends of linear frequencies. For example, consider

$$\dot{\theta} = 1 + \epsilon \sin(\theta - \phi) + \epsilon \cos(\theta + \phi) \tag{133}$$

$$\dot{\phi} = 1 + \delta + \epsilon \sin(\phi - \theta) + \epsilon \cos(\theta + \phi) \tag{134}$$

where $\delta \ll 1$. Subtracting the trend $\vartheta = \theta - t$ and $\varphi = \phi - t$ leads to

$$\dot{\vartheta} = \epsilon \sin(\vartheta - \varphi) + \epsilon \cos(\vartheta + \varphi - 2t) \tag{135}$$

$$\dot{\varphi} = \delta + \epsilon \sin(\varphi - \vartheta) + \epsilon \cos(\vartheta + \varphi - 2t) \tag{136}$$

Since $\dot{\varphi}$ and $\dot{\vartheta}$ are $O(\delta)$ by the averaging Theorem, fast oscillating terms containing $\cos$ are averaged out and can be neglected in a time scale as $1/\delta$. Thus, also in this case when performing a model recovery with finite amount of data the function U cannot be recovered. This also happens for our examples in the main text. Thus, sparsity and fast oscillations can contribute to the impossibility of recovering the original model.

Supplementary Note 9: Emergent hypernetworks in an integrate-and-fire model

Integrate and fire model. We used an autocatalytic integrate-and-fire model [5] to simulate the behavior of four oscillators in a ring configuration with state variable v_k, and a parameter for each oscillator p_k that determines whether the variable is increasing or decreasing. In the model, we introduce nonlinear time-delayed coupling, and the oscillators are governed by the equations

$$\frac{dv_k}{dt} = \frac{p_k v_k - (1-p_k) v_k B}{F_k} + p_k K \sum_{l=1}^{4} A_{k,l}(\tilde{v}_k + \tilde{v}_k^2)\tilde{v}_l(t-\tau) \tag{137}$$

where F_k is a rescaling factor that affects the natural frequency of kth oscillator, K is the coupling strength, $\tilde{v}_k$ is the signal corrected for offset ($\tilde{v}_k = v_k - 0.626$), $A_{k,l}$ is the adjacency matrix, and τ is the time delay.

When the variable v_k reaches 1 from below, then p_k is smoothly set to 0, and v_k decreases. Similarly, when the variable v_k reaches from above A, p_k is set to 1, and the variable starts to increase. We selected the threshold parameter $A = 0.36$ and the timescale parameter $B = 3.333$ so that only the one-cluster is stable with positive coupling. Then we adjusted the parameter F_k (F_1=4.950, F_2=1.955, F_3=3.177, F_4=1.970) of each oscillator to have a frequency ratio with respect oscillator 1 as $\omega_2/\omega_1 \approx 2.5$, $\omega_3/\omega_1 \approx 1.5$ and $\omega_4/\omega_1 \approx 2.5$. Note that F_k only affects the local dynamics of oscillator k and not the coupling term. Figure 9 (a) shows the time series of the variable v_k for K=0.234 and τ=1.65 s.

Fitting of phase dynamics. Similar to the experiments, we extract the phase of each oscillator using the peak-finding approach [1] from the time series of the variable v_k. When there is coupling and delay, the triplet phase differences, ϕ_j, j=1, 2, show a phase slip behavior Supplementary Fig. 9 (b). As described in the main text we used LASSO to fit the $\dot{\theta}_k$ values accoriding to equation (1) with drifting in the natural frequencies. The time series of the v_k variable (see Supplementary Fig. 9 (a)) showed a more nonlinear wave form, and we fitted the amplitudes of sin and cos until the seond order harmonics ($C^k_{j,2}$ and $D^k_{j,2}$). The $\dot{\theta}_k$ was filtered by a first order Savitzky-Golay filter for 125 s. Supplementary Fig. 9 (c) shows the corresponding fits for oscillator 1 to 4. (In the LASSO fit, we used a regularization parameter that represented an error 40% higher than the best fit). The fitted parameters are shown in Supplementary Fig. 10.

The strength of the triplet interactions on oscillator k is given by the amplitudes of the first and second harmonics (H^k_j); the amplitudes are shown in Supplementary Fig. 9d. In agreement with the experiments, the dynamics of oscillators 1 and 3 are impacted by both triplet interactions ϕ_1 and ϕ_2. For oscillator 1, the amplitudes are 1.7 $\times 10^{-3}$ and 1.0 $\times 10^{-3}$, and for oscillators 3 the amplitudes are 5.6 $\times 10^{-4}$ and 7.4 $\times 10^{-4}$ respectively. However, the dynamics of oscillator 2 and 4 are only impacted by ϕ_1 (2.1 $\times 10^{-3}$) and ϕ_2 (1.1 $\times 10^{-3}$).

We conclude that in an integrate-and-fire model, the phase dynamics of the oscillators coupled in a ring can be described by an emergent hypernetwork.

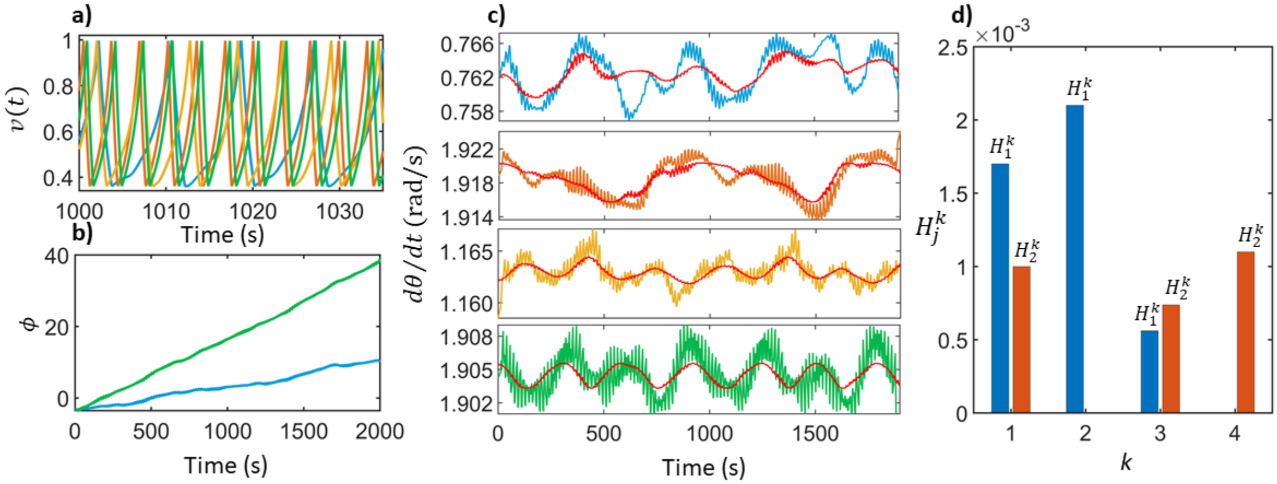

Supplementary Figure 9: **Simulations and network reconstruction with the integrate-and-fire model** a) Time series of the v_k variable. The blue, orange, yellow, and green lines correspond to oscillators one to four. b) Time series of the slow phases, ϕ_1 (blue) and ϕ_2 (green) with coupling and delay. c) The instantaneous frequency and the phase model fitted values (red) for oscillators 1 to 4 (corresponding from top to bottom). d) Coupling amplitudes for the four oscillators (k) from hypernetworks one (blue) and 2 (red).

Coefficients	Oscillator number			
	1	2	3	4
$\widehat{\omega}_k^0$	0.762	1.918	1.163	1.904
$\widehat{\omega}_k^1$	0	0	0	0
$\widehat{\omega}_k^2$	0	0	0	0
C_1^k	1.3×10^{-3}	-1.8×10^{-3}	5.0×10^{-4}	0
$C_{1,2}^k$	1.0×10^{-4}	7.0×10^{-4}	3.0×10^{-4}	0
D_1^k	7.0×10^{-4}	5.0×10^{-4}	0	0
$D_{1,2}^k$	9.0×10^{-4}	4.0×10^{-4}	0	0
C_2^k	-5.0×10^{-4}	0	7.0×10^{-4}	-1.1×10^{-3}
$C_{2,2}^k$	8.0×10^{-4}	0	1.0×10^{-4}	1.0×10^{-4}
D_2^k	0	0	0	0
$D_{2,2}^k$	0	0	0	0

Supplementary Figure 10: **Hypernetwork fitting coefficients for phase dynamics of the integrate-and-fire model.**

Supplementary Note 10: Mean field interaction

We consider the system

$$\dot{z}_k = \gamma_k z_k - \beta_k z_k |z_k|^2 + \alpha \sum_{\ell=1}^{n} A_{k\ell}(z_\ell + \bar{z}_\ell z_k^2)\,, \tag{138}$$

where A is the 4-ring network, with nodes labelled 1 through 4 along the ring. These frequencies satisfy the resonance conditions $\omega_1 + \omega_3 \approx 2\omega_2$ and $\omega_2 + \omega_4 \approx 2\omega_1$. A priori, it is unclear what the behavior of the system (138) will look like. To elucidate this, we conjugate the system by a transformation designed to get rid of the third order coupling terms in α. To this end, we define new coordinates

$$w_k = z_k - \alpha \sum_{\ell=1}^{n} \frac{A_{k\ell}}{\gamma_k + \bar{\gamma}_\ell} z_k^2 \bar{z}_\ell \,. \tag{139}$$

This causes new terms in α to appear, related to the $\beta_k z_k |z_k|^2$ terms in Equation (138). We therefore perform another coordinate transformation

$$u_k = w_k - \alpha Q_k(w) \,, \tag{140}$$

for some suitably chosen polynomials Q_k. We get equations for $\dot{u}_k$, which involve, among others, combinations of the linear and non-linear terms in the coupling

$$\alpha \sum_{\ell=1}^{n} A_{k\ell}(z_\ell + \bar{z}_\ell z_k^2) \,. \tag{141}$$

We then discard non-resonant terms in α^2, which leaves the equations

$$\begin{aligned}
\dot{u}_1 &= \gamma_1 u_1 - \beta_1 u_1 |u_1|^2 + \alpha(u_2 + u_4) + \alpha^2 \frac{u_2^2 \bar{u}_3}{\gamma_2 + \bar{\gamma}_3} + \text{h.o.t.} \\
\dot{u}_2 &= \gamma_2 u_2 - \beta_2 u_2 |u_2|^2 + \alpha(u_1 + u_3) + \alpha^2 \frac{u_1^2 \bar{u}_4}{\gamma_1 + \bar{\gamma}_4} + \text{h.o.t.} \\
\dot{u}_3 &= \gamma_3 u_3 - \beta_3 u_3 |u_3|^2 + \alpha(u_2 + u_4) + \alpha^2 \frac{u_2^2 \bar{u}_1}{\gamma_2 + \bar{\gamma}_1} + \text{h.o.t.} \\
\dot{u}_4 &= \gamma_4 u_4 - \beta_4 u_4 |u_4|^2 + \alpha(u_1 + u_3) + \alpha^2 \frac{u_1^2 \bar{u}_2}{\gamma_1 + \bar{\gamma}_2} + \text{h.o.t.} \,.
\end{aligned} \tag{142}$$

See Section SN 10.3 for more details on these normal form calculations. If we ignore the (non-resonant) terms $\alpha(u_2 + u_4)$ and $\alpha(u_1 + u_3)$ in Equation (142), then averaging yields the emergent phase dynamics. To this end, we set $\varphi_1 := \phi_1 - 2\phi_2 + \phi_3$ and $\varphi_2 := \phi_2 - 2\phi_1 + \phi_4$ for the slow phases. The different monomials in Equation (142) then yield terms in the phase equations according to:

- for node 1, $\frac{u_2^2 \bar{u}_3}{\gamma_2 + \bar{\gamma}_3}$ gives terms involving sin/cos of φ_1;
- for node 2, $\frac{u_1^2 \bar{u}_4}{\gamma_1 + \bar{\gamma}_4}$ gives terms involving sin/cos of φ_2;
- for node 3, $\frac{u_2^2 \bar{u}_1}{\gamma_2 + \bar{\gamma}_1}$ gives terms involving sin/cos of φ_1;
- for node 4, $\frac{u_1^2 \bar{u}_2}{\gamma_1 + \bar{\gamma}_2}$ gives terms involving sin/cos of φ_2.

SN 10.1 Frequency shifts

The linear terms $\alpha(u_2 + u_4)$ and $\alpha(u_1 + u_3)$ nevertheless have an effect on the emergent dynamics, in the following way. Whereas the natural frequencies of the uncoupled system (i.e. for $\alpha = 0$) are given by $\omega_1, \ldots, \omega_4$, they are in general given by the imaginary part of the eigenvalues of the perturbed matrix

$$U = \lambda I + i\Omega + \alpha A$$

where, Ω is the diagonal matrix with entries $\omega_1, \ldots, \omega_4$, and A is the adjacency matrix of the network. Eigenvalue perturbation then gives augmented frequencies of the form $\omega_k + O(\alpha^2)$. Note that the frequency perturbation is again of order α^2. This can be explained by a linear transformation bringing the perturbed system $\lambda I + i\Omega + \alpha A$ to that of the form $\lambda I + i\Omega + \alpha^2 B$, similar to our techniques for non-linear terms.

Therefore, whenever $\alpha > 0$ the frequencies will shift providing a frequency mismatch between the slow phases

$$\varphi_1 = \theta_1 - 2\theta_2 + \theta_3 \tag{143}$$

$$\varphi_2 = \theta_2 - 2\theta_1 + \theta_4 \tag{144}$$

namely, they will be modelled as

$$\dot{\varphi}_{1,2} = \varepsilon_{1,2} + G_{1,2}(\varphi_1) + H_{1,2}(\varphi_2). \tag{145}$$

where $\varepsilon_{1,2} = O(\alpha^2)$.

SN 10.2 Model Recovery

We integrate Eq. (138) with $\Omega_1 = 2, \Omega_2 = 3, \Omega_3 = 4$ and $\Omega_4 = 1$, by employing a wrapper of ODEPACK routine. Numerical integration was performed for 25000s with 0.01 time step. We discard the first 5000s points as transient.

We apply sparse regression using PySINDy Python package [6] with the Lasso optimizer on the phases θ_i considering the slow phases $\varphi_{1,2}$ with a penalty term $\lambda = 5 \times 10^{-3}$, we obtain

$$\dot{\theta}_1 = 2.001 + 0.018\cos(\varphi_1) \tag{146}$$

$$\dot{\theta}_2 = 2.999 - 0.015\cos(\varphi_2) \tag{147}$$

$$\dot{\theta}_3 = 3.992 - 0.011\cos(\varphi_1) \tag{148}$$

$$\dot{\theta}_4 = 1.008 + 0.011\cos(\varphi_2). \tag{149}$$

To recover the slow phase dynamics of φ_1 and φ_2 we apply the Lasso method with a penalty term $\lambda = 10^{-5}$ after applying a rolling window averaging process using window size of 100s to smooth

the fast oscillations to have better fit on slow phases. The obtained equation after the Lasso approach reads as

$$\dot{\varphi}_1 = -0.008 + 0.002\sin(\varphi_1) + 0.001\cos(\varphi_2) \tag{150}$$
$$\dot{\varphi}_2 = \quad 0.008 - 0.001\cos(\varphi_1) + 0.002\sin(\varphi_2). \tag{151}$$

The theory and the fitting are also in a perfect agreement for this mean-field case.

SN 10.3 Normal Form Calculations

Here we consider the case where we have both (non-resonant) linear coupling terms as well as higher order ones. More precisely, we consider the system

$$\dot{z}_k = \gamma_k z_k - \beta_k z_k |z_k|^2 + \alpha \sum_{\ell=1}^{n} c_{k,\ell} z_\ell + \alpha H_k(z)\,, \tag{152}$$

where H_k has only terms of degree 3 and higher. Later, we will set $H_k(z) = \sum_{\ell=1}^{n} c_{k,\ell} z_k^2 \bar{z}_\ell$. We assume that corresponding functions P_k exist that solve

$$\Gamma P_k - \gamma_k P_k = H_k\,. \tag{153}$$

In particular, when $H_k(z) = \sum_{\ell=1}^{n} c_{k,\ell} z_k^2 \bar{z}_\ell$ we assume that $\omega_k \neq \omega_\ell$ whenever $c_{k,\ell} \neq 0$, so that we may define

$$P_k(z) = \sum_{\ell=1}^{n} \frac{c_{k,\ell}}{\gamma_k + \bar{\gamma}_\ell} z_k^2 \bar{z}_\ell\,. \tag{154}$$

With slight abuse of notation, the sum in Equation (154) is taken over all ℓ such that $c_{k,\ell} \neq 0$. As before, we consider the coordinate transformation $w_k = z_k - \alpha P_k(z)$, which gives

$$z_k = w_k + \alpha P_k(w) + \alpha^2 [P_k||P](w) + \mathscr{O}(|\alpha|^3 |w|^7)\,, \tag{155}$$

by lemmas Supplementary Note 2:.18 and Supplementary Note 2:.21. A calculation as before reveals that

$$\begin{aligned}\dot{w}_k &= \gamma_k w_k - \beta_k w_k|w_k|^2 + \alpha\sum_{\ell=1}^{n} c_{k,\ell}w_\ell + \alpha(\gamma_k P_k(w) - \Gamma P_k(w) + H_k(w)) \\ &+ \alpha(L_k^1(w) - L_k^2(w)) + \alpha^2[\gamma_k P_k - \Gamma P_k + H_k||P](w) \\ &+ \alpha^2\sum_{\ell=1}^{n} c_{k,\ell}P_\ell(w) - \alpha^2[P_k||(\dots,\sum_{\ell=1}^{n} c_{j,\ell}w_\ell,\dots)](w) - \alpha^2[P_k||H](w) \\ &+ \mathscr{O}(|\alpha|^2|w|^7 + |\alpha|^3|w|^5) \\ &= \gamma_k w_k - \beta_k w_k|w_k|^2 + \alpha\sum_{\ell=1}^{n} c_{k,\ell}w_\ell + \alpha(L_k^1(w) - L_k^2(w)) \\ &+ \alpha^2\sum_{\ell=1}^{n} c_{k,\ell}P_\ell(w) - \alpha^2[P_k||(\dots,\sum_{\ell=1}^{n} c_{j,\ell}w_\ell,\dots)](w) - \alpha^2[P_k||H](w) \\ &+ \mathscr{O}(|\alpha|^2|w|^7 + |\alpha|^3|w|^5)\end{aligned} \tag{156}$$

where in the last step we have used Equation (153), and where we again set

$$\begin{aligned}&L_k^1(w) := [P_k||(\dots,\beta w_j|w_j|^2,\dots)](w) \text{ and} \\ &L_k^2(w) := [\beta_k w_k|w_k|^2||P](w)\,.\end{aligned} \tag{157}$$

Next, we perform a second transformation $u_k = w_k - \alpha Q_k(w)$, where Q_k solves

$$\Gamma Q_k - \gamma_k Q_k = L_k^1 - L_k^2\,, \tag{158}$$

and where

$$w_k = u_k + \alpha Q_k(u) + \mathscr{O}(|\alpha|^2|u|^9)\,. \tag{159}$$

This gives

$$\begin{aligned}\dot{u}_k &= \gamma_k u_k - \beta_k u_k|u_k|^2 + \alpha\sum_{\ell=1}^{n} c_{k,\ell}u_\ell + \alpha(\gamma_k Q_k(u) - \Gamma Q_k(u) + L_k^1(u) - L_k^2(u)) \\ &+ \alpha^2\sum_{\ell=1}^{n} c_{k,\ell}Q_\ell(u) - \alpha^2[Q_k||(\dots,\sum_{\ell=1}^{n} c_{j,\ell}u_\ell,\dots)](u) \\ &+ \alpha^2\sum_{\ell=1}^{n} c_{k,\ell}P_\ell(u) - \alpha^2[P_k||(\dots,\sum_{\ell=1}^{n} c_{j,\ell}w_\ell,\dots)](u) - \alpha^2[P_k||H](u) \\ &+ \mathscr{O}(|\alpha||u|^7 + |\alpha|^3|u|^5)\,.\end{aligned} \tag{160}$$

By Equation (158) we therefore get

$$\dot{u}_k = \gamma_k u_k - \beta_k u_k |u_k|^2 + \alpha \sum_{\ell=1}^{n} c_{k,\ell} u_\ell \tag{161}$$
$$+ \alpha^2 \sum_{\ell=1}^{n} c_{k,\ell} Q_\ell(u) - \alpha^2 [Q_k || (\dots, \sum_{\ell=1}^{n} c_{j,\ell} u_\ell, \dots)](u)$$
$$+ \alpha^2 \sum_{\ell=1}^{n} c_{k,\ell} P_\ell(u) - \alpha^2 [P_k || (\dots, \sum_{\ell=1}^{n} c_{j,\ell} w_\ell, \dots)](u) - \alpha^2 [P_k || H](u)$$
$$+ \mathscr{O}(|\alpha||u|^7 + |\alpha|^3 |u|^5) \,.$$

We now return to the special case of $H_k(z) = \sum_{\ell=1}^{n} c_{k,\ell} z_k^2 \bar{z}_\ell$. By assumption, the terms in α are resonant. Of the terms in α^2, only

$$\alpha^2 \sum_{\ell=1}^{n} c_{k,\ell} P_\ell(u) - \alpha^2 [P_k || (\dots, \sum_{\ell=1}^{n} c_{j,\ell} w_\ell, \dots)](u) \tag{162}$$

is third order in u (as opposed to fifth order). A direct calculation shows that

$$\sum_{\ell=1}^{n} c_{k,\ell} P_\ell(u) - [P_k || (\dots, \sum_{\ell=1}^{n} c_{j,\ell} w_\ell, \dots)](u) \tag{163}$$
$$= \sum_{\ell=1}^{n} \sum_{p=1}^{n} \frac{c_{k,\ell} c_{\ell,p}}{\gamma_\ell + \bar{\gamma}_p} u_\ell^2 \bar{u}_p - \sum_{\ell=1}^{n} \sum_{p=1}^{n} \frac{c_{k,\ell} c_{\ell,p}}{\gamma_k + \bar{\gamma}_\ell} u_k^2 \bar{u}_p - 2 \sum_{\ell=1}^{n} \sum_{p=1}^{n} \frac{c_{k,\ell} c_{k,p}}{\gamma_k + \bar{\gamma}_\ell} u_k \bar{u}_\ell u_p \,.$$

From these the resonant terms can be selected, which leads to a hypernetwork description of the dynamics.

References

[1] Pikovsky, A., Kurths, J., Rosenblum, M. & Kurths, J. *Synchronization: a universal concept in nonlinear sciences*, vol. 12 (Cambridge university press, 2003).

[2] Eroglu, D., Lamb, J. S. W. & Pereira, T. Synchronisation of chaos and its applications. *Contemporary Physics* **58**, 207–243 (2017).

[3] Stankovski, T., Pereira, T., McClintock, P. V. E. & Stefanovska, A. Coupling functions: Universal insights into dynamical interaction mechanisms. *Rev. Mod. Phys.* **89**, 045001 (2017).

[4] Novaes, M., Santos, E. R. d. & Pereira, T. Recovering sparse networks: Basis adaptation and stability under extensions. *arXiv preprint arXiv:2104.00796* (2021).

[5] Kori, H., Kiss, I. Z., Jain, S. & Hudson, J. L. Partial synchronization of relaxation oscillators with repulsive coupling in autocatalytic integrate-and-fire model and electrochemical experiments. *Chaos* **28**, 045111 (2018). URL `https://doi.org/10.1063/1.5022497`.

[6] Kaptanoglu, A. A. *et al.* Pysindy: A comprehensive python package for robust sparse system identification. *Journal of Open Source Software* **7**, 3994 (2022). URL `https://doi.org/10.21105/joss.03994`.
