# Supplementary material for: Emergent hypernetworks in weakly coupled oscillators
Source: Nat Commun. 2022 Aug 17;13:4849. doi: 10.1038/s41467-022-32282-4 (PMC9385626; doi:10.1038/s41467-022-32282-4)
Supplement: Supplementary file 3 — Description of additional Supplementary File [file 41467_2022_32282_MOESM3_ESM.pdf]

Descriptions of additional Supplementary files

Supplementary Software: Python codes and libraries to recover the hypernetworks from original network and isolated dynamics
